# Allele-specific expression reveals genes with recurrent cis-regulatory alterations in high-risk neuroblastoma

**DOI:** 10.1186/s13059-022-02640-y

**Published:** 2022-03-04

**Authors:** Arko Sen, Yuchen Huo, Jennifer Elster, Peter E. Zage, Graham McVicker

**Affiliations:** 1 Integrative Biology Laboratory, Salk Institute for Biological Studies, La Jolla, California USA; 2 Department of Pediatrics, Division of Hematology-Oncology, University of California San Diego, La Jolla, California USA; 3 Peckham Center for Cancer and Blood Disorders, Rady Children’s Hospital-San Diego, San Diego, California USA

## Abstract

**Background:**

Neuroblastoma is a pediatric malignancy with a high frequency of metastatic disease at initial diagnosis. Neuroblastoma tumors have few recurrent protein-coding mutations but contain extensive somatic copy number alterations (SCNAs) suggesting that mutations that alter gene dosage are important drivers of tumorigenesis. Here, we analyze allele-specific expression in 96 high-risk neuroblastoma tumors to discover genes impacted by cis-acting mutations that alter dosage.

**Results:**

We identify 1043 genes with recurrent, neuroblastoma-specific allele-specific expression. While most of these genes lie within common SCNA regions, many of them exhibit allele-specific expression in copy neutral samples and these samples are enriched for mutations that are predicted to cause nonsense-mediated decay. Thus, both SCNA and non-SCNA mutations frequently alter gene expression in neuroblastoma. We focus on genes with neuroblastoma-specific allele-specific expression in the absence of SCNAs and find 26 such genes that have reduced expression in stage 4 disease. At least two of these genes have evidence for tumor suppressor activity including the transcription factor *TFAP2B* and the protein tyrosine phosphatase *PTPRH*.

**Conclusions:**

In summary, our allele-specific expression analysis discovers genes that are recurrently dysregulated by both large SCNAs and other cis-acting mutations in high-risk neuroblastoma.

**Supplementary Information:**

The online version contains supplementary material available at 10.1186/s13059-022-02640-y.

## Background

Neuroblastoma is an extracranial solid tumor of the peripheral sympathetic nervous system which accounts for approximately 8% of all childhood cancers and 15% of childhood cancer mortality [1–6]. Compared to other pediatric malignancies, neuroblastomas harbor few recurrent somatic mutations, and most tumors lack identifiable driver mutations in protein-coding genes at the time of initial diagnosis [7]. Instead, neuroblastoma tumors are characterized by frequent somatic copy number alterations (SCNAs). The most common focal SCNA is amplification of the chromosome 2p24 region, including the *MYCN* oncogene, which is associated with high-risk disease and adverse treatment outcomes [8, 9]. Other common SCNAs span tens of megabases and include loss of distal chromosome arms 1p, 3p, and 11q and duplication of the distal arm of chromosome 17q [7, 9–12]. These large SCNAs may drive tumorigenesis by altering the expression of multiple tumor suppressors or oncogenes. For example, chromosome 1p deletions affect many potential tumor suppressors including *CHD5*, *CAMTA1*, *KIF1B*, *CASZ1*, *UBE4B*, and *MIR34A* [13–21]. In addition to the common SCNAs described above, neuroblastoma tumors also contain a patchwork of less common SCNAs or loss of heterozygosity (LOH) regions. A major challenge in interpreting large SCNAs is that they span dozens of genes, making it difficult to distinguish between driver and passenger genes.

Prior studies of genes with altered dosage in neuroblastoma have largely focused on functional characterization of genes affected by SCNAs while disregarding other dosage-altering mutations. Discovery of important driver genes dysregulated by non-SCNA mutations has been limited because only a small number of whole genome sequences for neuroblastoma are available, and it is difficult to determine which noncoding variants affect gene regulation. We hypothesized that genome-wide analysis of allele-specific expression (ASE) could illuminate dysregulated genes in neuroblastoma tumors.

**Fig. 1:**
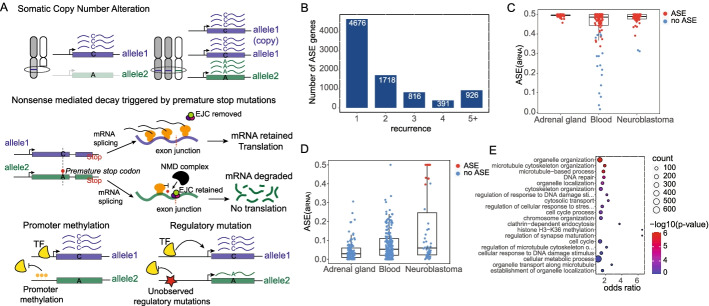
Allele-specific expression analysis in neuroblastoma. **A** Four example mechanisms that can cause allele-specific expression (ASE) of genes: (i) somatic copy number alterations (deletions or duplications), (ii) premature stop mutations that trigger nonsense mediated decay (NMD), (iii) promoter DNA methylation, and (iv) cis-acting mutations that affect gene regulatory sequences such as enhancers. These examples are not exhaustive and there are additional genomic alterations that can cause ASE. **B** Recurrence of ASE across 96 neuroblastoma samples for genes with ASE in at least one sample (FDR < 0.1). **C** Estimates of ASE (a_RNA_) for *H19*, an established imprinted gene, in neuroblastoma tumor samples compared to normal adrenal gland and whole blood samples from GTEx. **D** Estimates of ASE for *KIF1B*, a known neuroblastoma tumor-suppressor gene. **E** Gene Ontology analysis of 1043 genes with recurrent ASE in neuroblastoma (NB-ASE genes)

ASE quantifies the difference in expression of two alleles of a gene and can be measured using RNA-seq reads that align to heterozygous sites. Compared to standard differential gene expression analysis, ASE is insensitive to environmental or trans-acting factors, which generally affect both alleles equally. This makes ASE a powerful tool for revealing genes that are affected by cis-acting mutations, including noncoding regulatory mutations that affect sequences such as promoters, enhancers, and insulators as well as protein-coding or splicing mutations that result in nonsense-mediated decay (NMD) (Fig. 1A). Another advantage of ASE is that it is detectable even when the identity of the pathogenic variants causing dysregulation is unknown and it can reveal the effects of rare germline or somatic mutations [22, 23]. Thus, ASE is a powerful tool for the identification of genes with altered gene dosage due to cis-acting genome alterations.

In addition to somatic mutations, ASE can also be caused by common germline polymorphisms [24–26], imprinting [27] or random monoallelic expression [28, 29]; however, these factors are less likely to be involved in tumorigenesis. To discover genes which are dysregulated by pathogenic events, ASE in disease tissue can be compared to either paired-normal tissue or to a large panel of normal tissues to identify cancer-specific gene dysregulation [22, 23, 30, 31]. Genome-wide analysis of ASE therefore has the potential to reveal novel tumor suppressor and oncogenes both within and outside SCNAs.

## Results

To discover genes with ASE in neuroblastoma tumors, we obtained exome-seq and RNA-seq data for 96 neuroblastoma tumor samples from the NCI Therapeutically Applicable Research to Generate Effective Treatments (TARGET) project. To estimate ASE in these samples, we implemented a statistical model that utilizes allele-specific read counts at heterozygous sites within the exons of genes, while accounting for genotyping errors, sequencing errors, and overdispersion of RNA-seq read counts. This model estimates allele imbalance (*a*_*RNA*_) for each gene, which is how far the reference allele proportion differs from the expected value of 0.5.

With this method, we identify 8527 genes with ASE in at least one tumor sample under a false discovery rate (FDR) of 10% (likelihood ratio test). Most genes exhibit ASE in only a single sample (4676 out of 8527); however, 3851 genes have ASE in more than one sample, and many genes show highly recurrent ASE in neuroblastoma (926 genes have ASE in 5 or more samples) (Fig. 1B and Additional file 2: Table S1). Since recurrent ASE can result from non-pathogenic factors including common germline polymorphisms [24–26], imprinting [27], or random monoallelic expression [28, 29, 32], we compared the frequency of ASE in neuroblastoma to that of normal tissues, obtained from the genotype tissue expression project (GTEx). Specifically, we compared neuroblastoma ASE estimates to those from normal adrenal gland and whole-blood tissues. These tissues were chosen because the adrenal cortex is the tissue of origin for most neuroblastoma tumors and whole-blood has by far the largest number of available samples in GTEx. To illustrate the utility of comparing normal and tumor tissues, we examined ASE for a well-established imprinted gene, *H19* [33], and for a tumor suppressor gene, *KIF1B*, which is located on chromosome 1p and is frequently deleted in neuroblastoma [16, 34]. As expected, *H19* has very strong ASE in almost all normal and tumor samples (Fig. 1C), whereas ASE of *KIF1B* is observed exclusively in neuroblastoma samples (Fig. 1D).

To define a set of genes with neuroblastoma-specific ASE (NB-ASE), we used two filtering criteria: (a) genes that are testable for ASE in at least 10 neuroblastoma and 10 adrenal gland or blood samples and (b) genes with significant ASE in ≥ 3 neuroblastoma tumors and ≤1 normal tissue (Additional file 2: Table S2). These criteria resulted in 1043 NB-ASE genes for downstream analysis. We performed a Gene Ontology analysis of these genes and found that they are enriched in biological processes frequently dysregulated during tumorigenesis including microtubule-based process (GO:0007017, *p* value = 2.6e−06), DNA repair (GO:0006281, *p*-value = 2.5e−05), and cellular metabolic process (GO:0044237, *p*-value = 0.00074) (Fig. 1E and Additional file 2: Table S3).

**Fig. 2:**
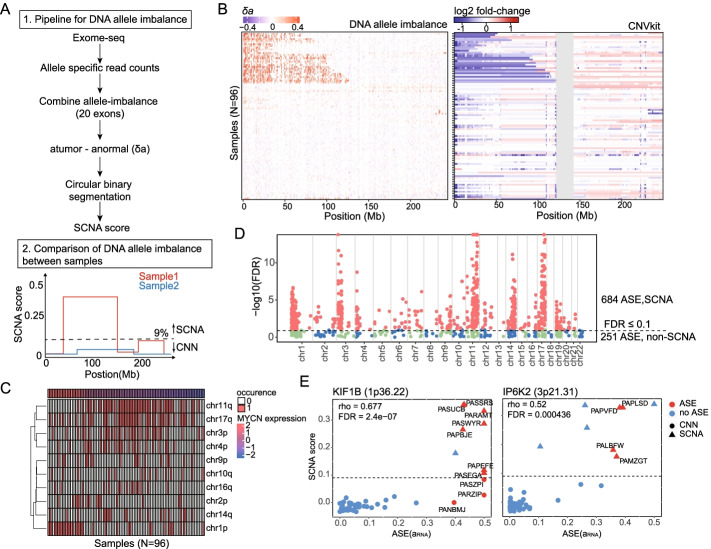
Detecting somatic copy number alterations in neuroblastoma. **A** Schematic of the DNA allelic imbalance method for detecting somatic copy number alterations (SCNAs). **B** Difference in DNA allelic imbalance between normal and tumor tissues (*δ*_*a*_) for 96 neuroblastoma patients across chromosome 1 (left panel). Results are compared to log2 fold-change in normalized read coverage between tumor and normal tissues estimated by CNVkit (right panel). **C** Occurrence matrix of the 10 most frequent SCNAs detected using the DNA-imbalance approach. SCNAs were filtered using SCNA score ≥ 0.09 and annotated based on their cytoband location. Neuroblastoma patients are grouped by *MYCN* z-score normalized gene expression. **D** Manhattan plot for Spearman’s correlation analysis between ASE (*a*_*RNA*_) and SCNA score for 945 NB-ASE genes. **E** Spearman’s rank correlation between allele-specific expression (*a*_*RNA*_) and SCNA score for *KIF1B* (left panel), a tumor suppressor in the chromosome 1p deletion region, and *IP6K2* (right panel), a putative tumor suppressor within the 3p deletion region

SCNAs are a common cause of ASE in tumors [35], and we hypothesized that many NB-ASE genes would be attributable to large-scale SCNAs that dominate the genetic landscape of neuroblastoma [2, 3, 6, 7]. To determine which NB-ASE genes can be attributed to SCNAs, we adapted our ASE framework to identify SCNAs, which are detectable as large genome segments with allelic imbalance of DNA sequencing reads [36]. While several existing tools leverage read depth to predict SCNAs, these methods have limited precision and report many false positive focal SCNAs [37, 38]. To detect SCNAs, we estimated DNA allelic imbalance from heterozygous sites in windows consisting of 20 consecutive exons for tumor (a_tumo*r*_) and normal (a_normal_) samples. We then computed the difference in their absolute values (*δ*_*a*_) and performed circular binary segmentation (CBS) [39] to obtain DNA allelic imbalance for continuous segments which we refer to as the SCNA score (Fig. 2A and Additional file 2: Table S4).

To test our allelic imbalance approach for SCNA discovery, we applied it to chromosome 1, which has distal p arm deletions in ~30% of neuroblastoma tumors [7, 10, 12]. We compared our predictions to those made by CNVkit, which utilizes read depth at exome capture targets to infer copy number and has better sensitivity compared to other methods for SCNA discovery [40]. SCNA breakpoints detected by our method are consistent with those detected by CNVkit, but our predictions are considerably less noisy (Fig. 2B). We also compared our results to those from high density single-nucleotide polymorphism (SNP) arrays, which were available for 33 out of the 96 neuroblastoma tumors [41], and found them to be highly concordant (Additional file 1: Fig. S1). We obtained similar results for other common SCNAs such as the chromosome 11q deletion region (Additional file 1: Fig. S2).

To further examine the SCNAs in neuroblastoma, we partitioned samples based on *MYCN* expression and found known patterns of SCNA co-occurrence [7]. For example, chromosome 1p and 11q deletions occur most frequently in samples with high and low expression of *MYCN*, respectively (Fig. 2C). In addition to the well-characterized SCNAs, we detected less frequent SCNAs across all chromosomes (Fig. 2C) including loss of 16q in 16 neuroblastoma tumors (Additional file 1: Fig. S3). This SCNA has not been extensively studied but has been previously reported by comparative genomic hybridization in familial neuroblastomas and some other pediatric cancers such as Wilm’s tumor [42–44]. Our deletion predictions for 16q appear to be true positives because they are concordant with both SNP-array predictions and patterns of ASE (Additional file 1: Fig. S3). In combination, these results indicate that DNA allelic imbalance is a powerful approach for the detection of SCNAs in cancer genomes.

To determine whether general patterns of ASE in neuroblastoma can be attributed to SCNAs, we computed Spearman’s correlation between ASE and SCNA score, restricting our analysis to 935 NB-ASE genes that are located within SCNA segments in at least one neuroblastoma sample. Under an FDR of 10%, 65% (684 out of 1043) of NB-ASE genes are significantly correlated with SCNAs, and of these, 59% (401 out 684) are located on the chromosomes with the most frequent SCNAs (chromosomes 1, 3, 11, and 17) (Fig. 2D and Additional file 2: Table S5).

The chromosome 1p, 3p, and 11q deletion regions are hypothesized to contain tumor suppressor genes; however, the identities of the tumor suppressors are difficult to determine because the deletions are large and contain hundreds of genes. We reasoned that, in the absence of large deletions, tumor suppressors within these regions may be affected by other types of genome alterations that affect dosage, and that the effects of these alterations would be detectable by ASE. We examined the relationship between ASE and SCNAs for two genes, *IP6K2* and *KIF1B*, which have been previously identified as potential tumor suppressors located within the chromosome 3p and 1p deletion regions. Knockdown of *IP6K2* impairs apoptosis in colorectal cancer cells [45], and its deletion or low expression is associated with adverse clinical outcomes in aerodigestive tract carcinoma and breast cancer [46, 47]. Overexpression of *KIF1B* in neuroblastoma cell lines causes apoptotic cell death and its knockdown enhances tumor formation in mouse models [34]. In the case of *IP6K2*, we found that every sample with significant ASE also has a high SCNA score (Fig. 2E, right panel), indicating that ASE of *IP6K2* is solely attributable to overlapping chromosome 3p deletions. The pattern exhibited by *KIF1B* is different. While ASE of *KIF1B* is correlated with SCNA score (Spearman’s rho = 0.68, FDR corrected *p*-value = 2.4e−07), several samples have strong ASE in the absence of a chromosome 1p deletion (SCNA score ≤ 0.09) (Fig. 2E, left panel). Thus, in some samples, ASE of *KIF1B* is caused by factors other than large-scale SCNAs. Several other putative tumor suppressors in the chromosome 1p or 11q deletion regions including *CHD5* [15, 48], *UBE4B* [13], *CADM1* [49], and *ATM* [50], have patterns that are similar to *KIF1B*, where a subset of samples exhibit strong ASE in the absence of deletions (Additional file 1: Fig. S4). These results indicate that both SCNA and non-SCNA genome alterations affect the expression of these genes.

**Fig. 3:**
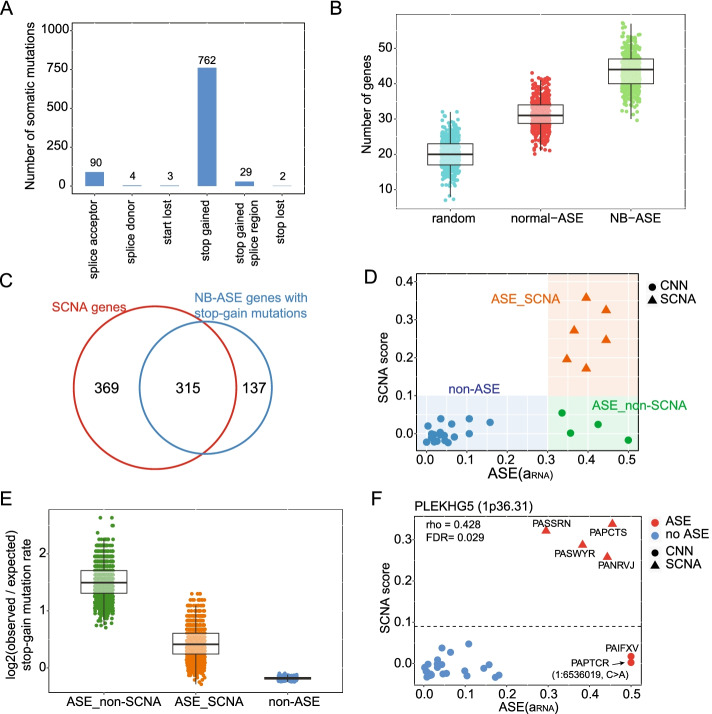
Genes with recurrent allele-specific expression are enriched for stop-gain mutations that cause nonsense-mediated decay. **A** The number of variants annotated as high-impact by variant effect predictor (VEP) in genes with recurrent allele-specific expression in neuroblastoma (NB-ASE genes) grouped by functional consequence. **B** NB-ASE genes more frequently carry stop-gain mutations. To create the distributions, we performed 500 sampling iterations and counted the number of unique genes carrying stop-gain mutations each iteration. In each iteration, we sampled 100 genes from three gene sets: a random panel of control genes (random), genes with ASE in both neuroblastoma and normal tissues (normal-ASE), and genes with ASE that is specific to neuroblastoma (NB-ASE). **C** Overlap between genes with correlated ASE and somatic copy number alteration (SCNA) score and NB-ASE genes which contain at least one stop-gain mutation. **D** Graphical representation of three categories of neuroblastoma samples we define for a given gene: samples without ASE (non-ASE), samples with ASE and SCNA (ASE_SCNA), and samples with ASE but no SCNA (ASE_non-SCNA). **E** Samples with ASE in the absence of SCNAs are enriched for stop-gain mutations. The boxplots show the log2 ratio of observed to expected stop-gain mutation rate for the three sample categories. Expected rates are estimated by permuting category labels within each gene; points are observed/expected ratios computed across genes after each permutation. **F** Spearman’s correlation between ASE (a_RNA_) and SCNA score for *PLEKHG5*, a chromosome 1p deletion gene. Two samples have high ASE but low SCNA scores and one of these samples, PAPTCR, carries a premature stop mutation (1:6536019, C>A)

Previous studies have demonstrated that ASE can be caused by nonsense-mediated decay (NMD) [51–53], an evolutionarily conserved mechanism that degrades transcripts with premature termination codons [54–56] (Fig. 1A). We hypothesized that genes that exhibit ASE in neuroblastoma in the absence of SCNAs may contain mutations that cause NMD. To identify somatic mutations that are likely to cause NMD, we analyzed paired tumor-normal exome-seq data with variant effect predictor (VEP), which collectively labels missense, frameshift, and nonsense mutations that are likely to cause NMD as “high-impact.” We identified 12,122 unique high-impact mutations in the 96 tumor samples, 886 of which are located within 490 NB-ASE genes. Most of these mutations (788 out of 890) are stop-gain mutations (Fig. 3A and Additional file 2: Table S6) and map to 452 NB-ASE genes.

To determine if stop-gain mutations are enriched within NB-ASE genes, we examined their frequency in three different gene sets: (a) NB-ASE genes, (b) randomly selected genes with at least one somatic mutation, and (c) genes with ASE observed in both neuroblastoma and normal tissues. To create a null distribution, we sampled 100 genes from each gene set 500 times and counted the number of genes carrying stop-gain mutations each sampling iteration. A substantially greater number of NB-ASE genes carry stop-gain mutations, indicating that NMD is an important driver of neuroblastoma-specific ASE (Fig. 3B).

We observed that many genes with correlated ASE and SCNA scores also contain stop-gain mutations in some samples (Fig. 3C), leading us to hypothesize that NMD is an important mechanism that alters gene dosage in samples lacking SCNAs. To test this hypothesis, we partitioned neuroblastoma samples for each gene into three categories: non-ASE samples, ASE samples with SCNAs (ASE_SCNA), and ASE samples without SCNAs (ASE_non-SCNA) (Fig. 3D). We then calculated the rate of stop-gain mutations across all genes and samples in each of the three categories. To generate a null distribution of rates that controls for gene lengths and mutation rate heterogeneity, we permuted the category labels for each gene 1000 times. This analysis revealed that stop-gain mutations occur at a substantially higher rate in ASE_non-SCNA samples compared to other categories (Fig. 3E). This supports the hypothesis that gene expression is often altered by NMD-causing mutations in the samples that lack SCNAs.

An example of a gene which is dysregulated by both NMD and SCNAs is Pleckstrin Homology and RhoGEF Domain Containing G5 (*PLEKHG5*). *PLEKHG5* is located in cytoband 1p36.31 which is frequently deleted in neuroblastoma [57]. Two samples have strong ASE in the absence of SCNAs, one of which is heterozygous for a C>A mutation that introduces a premature stop codon (Fig. 3F). The cause of ASE in the other ASE_non-SCNA sample is unknown and could potentially be discovered by analysis of whole-genome sequencing data.

Outside of SCNAs, 108 NB-ASE genes are located in genome regions that are copy neutral across all tumor samples, including *PHOX2B* which is a target of recurrent germline mutations in neuroblastoma [58, 59]. In addition, 251 genes lack significant correlations between ASE and SCNA score even though they overlap SCNAs in one or more samples (Additional file 2: Table S5). Thus, 34% of the NB-ASE genes (359 out of 1043) are not associated with SCNAs, suggesting that other mutational events that alter gene dosage are common in neuroblastoma genomes.

**Fig. 4:**
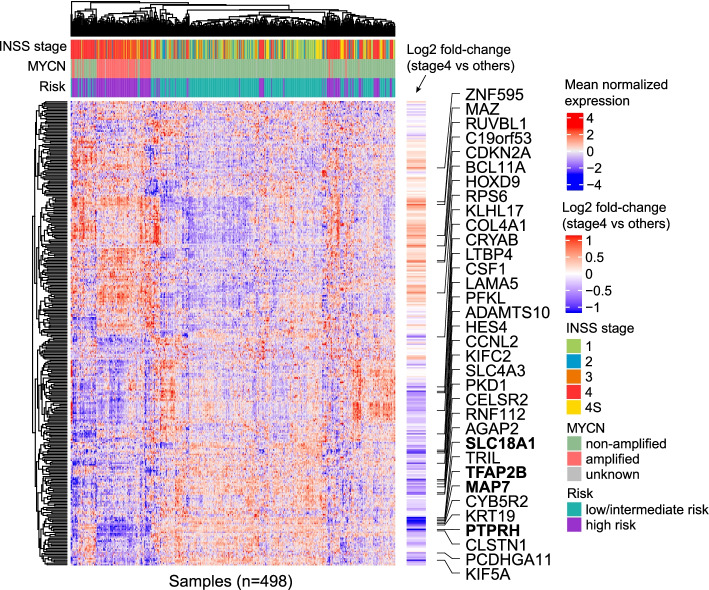
Expression of genes with recurrent allele-specific expression that is not associated with somatic copy number alterations. The heatmap shows hierarchically clustered gene expression z-scores from 498 neuroblastoma tumors in the SEQC/MAQC-III Consortium. Of the 359 NB-ASE genes that were not associated with somatic copy number alterations (SCNAs), 316 genes were covered by at least one expression probe. Samples are labeled by clinical characteristics: *MYCN* amplification status (amplified, non-amplified and unknown); high-risk tumor (yes or no); International Neuroblastoma Staging System (INSS) (1, 2, 3, 4, and 4S). Difference in gene-expression between stage 4 and all other stages is indicated as log2 fold-change (log2FC). The 34 genes with absolute log2FC ≥ 0.5 and FD-corrected *p*-value ≤ 0.05 from Student’s *t* test are labeled

We reasoned that the overall expression of NB-ASE genes could be examined in a larger gene expression dataset where ASE measurements are unavailable. We asked whether the 316 non-SCNA ASE genes are associated with neuroblastoma progression and metastasis, by analyzing the SEQC/MAQC-III Consortium dataset, which contains clinical and microarray expression data for 498 neuroblastoma tumors [60]. Under an FDR of 5% (Student’s *t* test) and absolute log2 fold-change ≥ 0.5, 34 genes have significantly different gene-expression in stage 4 or metastatic disease compared to other stages (Fig. 4). Among them, 8 genes have increased expression and 26 genes have decreased expression in stage 4 disease. Most notably, *MAP7*, *PTPRH, TFAP2B*, and *SLC18A1* have more than a 2-fold decrease in expression in stage 4 tumors. We hypothesized that these genes may be important tumor suppressors in neuroblastoma, even though they lie outside of the common SCNA regions of the genome, and we performed further functional analysis of *TFAP2B* and *PTPRH*.

We first investigated *TFAP2B*, which is a retinoic acid-induced transcriptional activator that mediates noradrenergic neuronal differentiation of neuroblastoma cells in vitro [61, 62]. *TFAP2B* has ASE in 3 out of 31 testable neuroblastoma samples, has no evidence of ASE in adrenal gland tissues (0 out of 12 testable samples), is not expressed in whole blood, and is copy neutral in all patient samples (Additional file 1: Fig. S5A). Consistent with the above observations, the samples with ASE of *TFAP2B* have strong allelic imbalance of RNA-seq reads at heterozygous sites, but no allelic imbalance of exome-seq reads, indicating that the ASE is not due to SCNAs (Additional file 1: Fig. S5B). Dysregulation of *TFAP2B* in neuroblastoma cells has previously been associated with aberrant promoter-methylation [62], so we investigated DNA methylation as a potential mechanism. Using estimates of promoter methylation computed from the Human Methylation 450K array, we found that *TFAP2B* is one of the NB-ASE genes with the strongest correlations between ASE and promoter-methylation, although this correlation is not significant under an FDR threshold of 10% (Spearman’s correlation coefficient = 0.60, FDR-corrected *p*-value = 0.116) (Additional file 1: Figs. S5C-E, S6 and Additional file 2: Table S7). Furthermore, one patient sample (PASNZU) has near-complete methylation (>75%) of the *TFAP2B* promoter, which is associated with loss-of-expression of both alleles (Additional file 1: Fig. S5C-E and Additional file 2: Table S8). In the SEQC/MAQC-III Consortium data, *TFAP2B* expression is decreased in stage 4 or metastatic neuroblastomas (Additional file 1: Fig. S5F) and low expression of *TFAP2B* is associated with worse event-free survival outcomes in non-*MYCN* amplified neuroblastoma patients (Additional file 1: Fig. S5G). Collectively these observations are consistent with earlier findings [62] and strongly suggest that *TFAP2B* is a tumor-suppressor in neuroblastoma with decreased expression in the presence of promoter methylation.

**Fig. 5:**
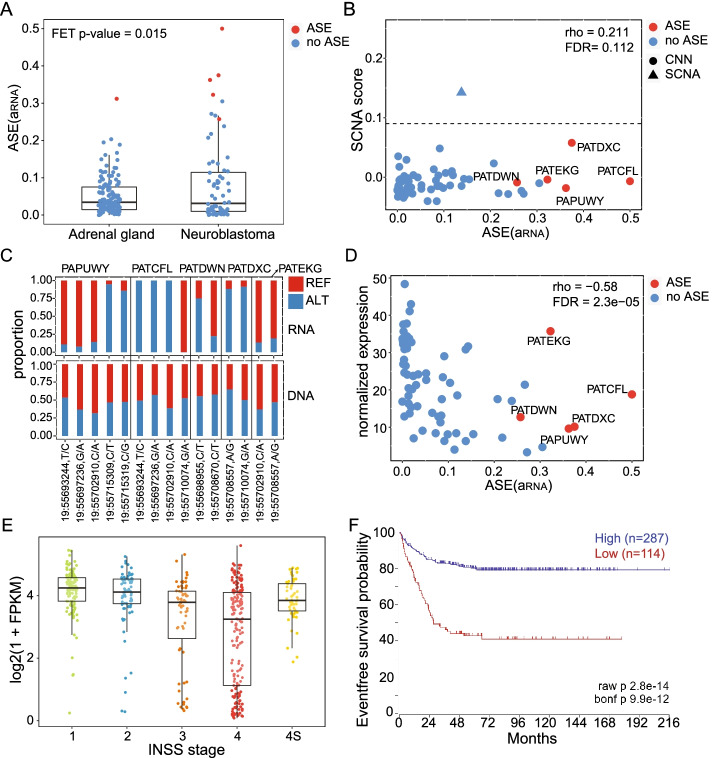
Genomic profiling of *PTPRH*. **A** ASE (*a*_*RNA*_) of *PTPRH* in neuroblastoma and adrenal gland tissues. *PTRPH* has detectable ASE in 5 out of 68 neuroblastoma patients and 1 out of 139 Adrenal gland tissues (Fisher’s exact test *p*-value = 0.015). **B** Spearman’s correlation between ASE (a_RNA_) for *PTPRH* and SCNA score for the overlapping genomic segments. **C** Reference and alternate allele proportions for RNA-seq and exome-seq reads at heterozygous sites within the *PTPRH* gene in 5 samples with significant ASE. **D** Spearman’s correlation between ASE (*a*_*RNA*_) and gene expression for *PTPRH.* **E** Normalized gene expression of *PTPRH* across different disease stages for 498 neuroblastoma patients from the SEQC/MAQC-III Consortium data. **F** Kaplan-Meier survival analysis comparing MYCN non-amplified patients with high or low expression of *PTPRH* from the SEQC/MAQC-III Consortium data set

**Fig. 6:**
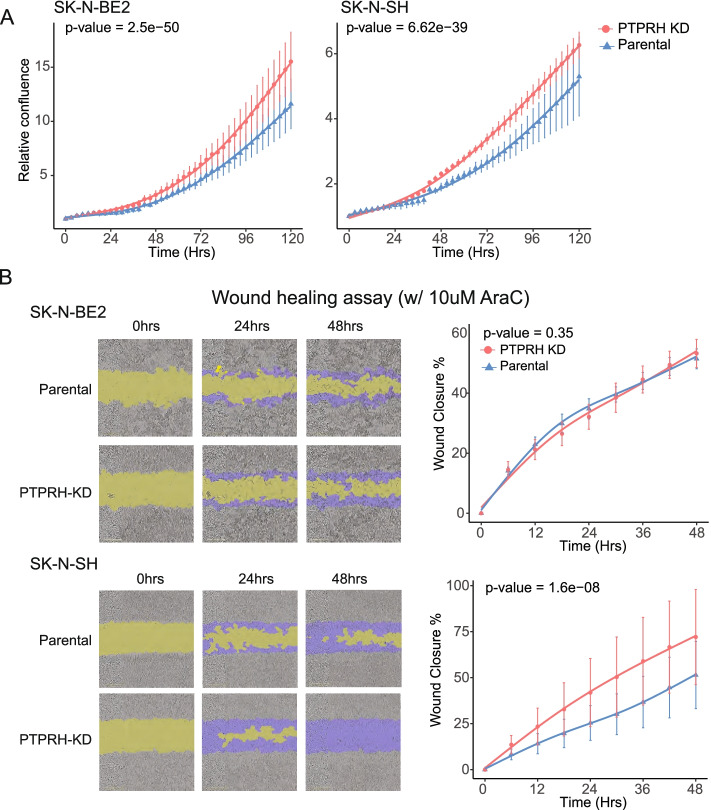
PTPRH knockdown in neuroblastoma cell lines increases cellular proliferation and migration. **A** Change in relative confluence (i.e., confluence at each time point normalized to confluence at time 0) for parental and PTPRH knockdowns (KDs) in SK-N-BE(2) and SK-N-SH cells. Six replicates for each condition were used for the SK-N-BE(2) cell line and SK-N-SH cell line. **B** Left panels show representative images for scratch wound assays used to measure cellular migration for parental and PTPRH-KD in SK-N-BE(2) and SK-N-SH cells. Right panels show wound closure percentage for parental and PTPRH-KD in SK-N-BE(2) and SK-N-SH cells over 48 h. Each time point had 9 replicates. Error bars indicate +/− one standard deviation around the mean across replicates at each time point. The lines are fits from cubic spline regression using 3 knots, and the *p*-value is from an F-test for a difference in splines between parental and KD cells

We next investigated *PTPRH* (Protein Tyrosine Phosphatase Receptor Type H), which is a member of a large family of receptor tyrosine phosphatases and a critical regulator of apoptosis and cell motility [63, 64]. In neuroblastoma, 5 out of 68 testable samples exhibit ASE, compared to 1 out 139 testable adrenal gland tissues from GTEx (Fisher’s exact test *p*-value = 0.015) (Fig. 5A); *PTPRH* is not expressed in the whole blood. *PTPRH* is located on chromosome 19q, which rarely undergoes SCNA in neuroblastoma, and ASE of *PTPRH* is not correlated with SCNA score (Fig. 5B). In addition, the RNA-seq reads in ASE samples exhibit strong allelic imbalance but there is no allelic imbalance in exome-seq reads, confirming that ASE of *PTPRH* is not attributable to large or focal SCNAs (Fig. 5C). ASE of *PTPRH* is negatively correlated with gene-expression (Spearman’s correlation coefficient = −0.58, FDR-corrected *p*-value = 2.3e−05) indicating that ASE reflects loss of expression of one allele, potentially due to regulatory or other cis-acting mutations (Fig. 5D and Additional file 2: Table S8). Gene expression of *PTPRH* is substantially reduced in stage 4 tumors, and reduced expression of this gene is associated with worse event-free survival outcomes in non-MYCN amplified tumors (Fig. 5E, F). To further test the function of *PTPRH*, we performed shRNA knockdown experiments in the neuroblastoma cell lines SK-N-SH and SK-N-BE(2), which are *MYCN* non-amplified and amplified, respectively (Additional file 1: Fig. S7). Knockdown of *PTPRH* increases proliferation in both cell lines and cellular migration in the SK-N-SH cell line (Fig. 6A, B). These results support the hypothesis that *PTPRH* is a MYCN-independent tumor suppressor.

## Discussion

Our study leveraged allelic imbalance of RNA and DNA sequencing reads to discover genes with recurrent ASE and delineate SCNA regions in neuroblastoma genomes. Neuroblastoma genomes contain a surprisingly large number of genes with recurrent ASE; however, the majority of ASE events can be attributed to SCNAs which are well-characterized and common genomic alterations in neuroblastoma that typically span tens of megabases.

Our ASE analysis revealed that, in some samples, genes within recurrent SCNA regions are dysregulated by non-SCNA events. Non-SCNA ASE events are present in genes which have been previously described as putative tumor suppressors and studied in the context of recurrent SCNAs including *KIF1B, PLEKHG5*, *UBE4B*, *CHD5*, *CADM1*, and *ATM*. With larger sample sizes, ASE could potentially be utilized to distinguish passenger genes from driver genes within recurrent SCNA regions. In some samples, ASE in the absence of SCNAs can be attributed to mutations that cause NMD; however, in other samples, the cause is unknown. In these cases, the cause of ASE could potentially be revealed by future studies. For example, whole genome sequencing and analysis of non-coding mutations near NB-ASE genes could illuminate cis-acting regulatory mutations that cause ASE.

Outside of recurrent SCNA regions, we discovered 359 genes that are recurrently dysregulated in neuroblastoma. These genes include *TFAP2B*, *MAP7*, *PTPRH*, and *SLC18A1*, which have substantially lower expression in stage 4 disease. *TFAP2B* is important for noradrenergic neuronal differentiation of neuroblastoma cells in vitro and is dysregulated by aberrant promoter methylation [62]. Our independent validation of this finding is additional evidence that *TFAP2B* is an important tumor suppressor in neuroblastoma. *PTPRH* belongs to a group of receptor tyrosine phosphotases which reduce phosphorylation of Akt and its cellular substrates such GSK-3α or GSK-3β [64]. *PTPRH* may inactivate Akt and promote apoptosis in cancer cells. In addition, overexpression of *PTPRH* has been demonstrated to disrupt actin-based cytoskeleton as well as inhibit cellular responses promoted by integrin-mediated cell adhesion, including cell spreading on fibronectin, growth factor-induced activation of extracellular signal-regulated kinase 2, and colony formation [63]. In our study, we found that (a) *PTPRH* exhibits recurrent ASE in neuroblastoma, (b) low expression of *PTPRH* is associated with adverse patient outcomes, and (c) knockdown of *PTPRH* increases proliferation and wound healing in neuroblastoma cell lines. Collectively, these observations suggest that *PTPRH* functions as a tumor suppressor in high-risk neuroblastomas. Confirmation that *PTPRH* acts as a tumor suppressor will require in vivo experiments that are beyond the scope of this study.

## Conclusions

In summary, our study provides a framework for analysis of ASE and SCNAs in tumors. Using this framework, we study the impact of genomic alterations that affect gene expression in neuroblastoma and discover that multiple types of mutations work in concert to dysregulate gene expression. While most ASE in neuroblastoma is driven by large-scale SCNAs, many genes exhibit ASE in samples that lack SCNAs. These samples are enriched for mutations that are predicted to cause NMD. In addition, we identify some genes that have recurrent ASE outside of common SCNA regions, including *TFAP2B* and *PTPRH*, both of which have low expression in stage 4 disease and evidence for tumor suppressor activity.

## Methods

### Datasets

Next-generation sequencing (NGS) data for neuroblastoma patients were obtained from the Therapeutically Applicable Research to Generate Effective Treatments (TARGET) initiative [7]. Our dataset consisted of RNA sequencing for 143 tumors and paired tumor-normal exome sequencing for 97 neuroblastoma patients. Out of the 97 samples with both RNA-seq and exome-seq data, 87 also had Illumina Infinium Human Methylation 450K data and 33 had HumanHap 550K BeadChIP (SNP-array) data. We also obtained 175 adrenal gland and 369 whole blood samples from the GTEx Consortium and used them as a normal ASE reference set [65].

### Quality control

To ensure that NGS data from the same patient are properly paired, we compared the RNA-seq and exome-seq data from 97 neuroblastoma tumors using NGSCheckMate [66]. Based on this analysis, we found that one sample had mismatched RNA-seq and exome-seq data and we removed this sample from the study. Our final dataset for ASE analysis consisted of RNA-seq and exome-seq data from 96 neuroblastoma patients.

### Variant calling pipeline

We aligned exome-seq reads to the reference genome (hg19) using BWA-MEM with default parameters [67]. Then, we generated GVCF files for each sample using the GATK *HaplotypeCaller* (4.1.1) and performed joint genotyping using GATK *GenotypeGVCFs*. We extracted single nucleotide polymorphism (SNPs) using GATK *SelectVariants* command and recalibrated variant quality scores with GATK variant quality score recalibration (VQSR) pipeline. The filtered and processed SNPs were used for downstream analyses.

### Somatic mutation discovery pipeline

We used Mutect2 from GATK (4.1.1) to compare the mutation profile from exome-seq data for 96 neuroblastoma tumor and normal whole blood samples [68]. We filtered somatic mutations from Mutect2 using the GATK recommended filtering pipeline (https://gatk.broadinstitute.org/hc/en-us/articles/360035531132). To determine the functional consequence of somatic mutations and to assign mutations to respective genes, we further analyzed individual somatic mutations in each sample using variant effect predictor (VEP) [69].

### Read depth-based detection of somatic copy number alternations

SCNAs in neuroblastoma were detected using our DNA allelic imbalance method described below and a read depth-based method, CNVkit [40]. Briefly, CNVkit uses exome-seq reads to calculate log2 copy ratios across the genome for tumor-normal pairs. SCNAs for large chromosomal regions are then detected by combining log2 copy ratios across adjacent genomic regions using Circular Binary Segmentation (CBS). For this study, we processed aligned exome-seq reads with the *batch* option from CNVkit using the *--drop-low-coverage* parameter to control for low coverage exome targets. Heatmaps showing CNV calls for all samples were generated using CNVkit’s *heatmap* script.

### RNA-seq alignment and processing

We aligned RNA-seq reads end-to-end to the reference genome (hg19) using STAR (2.5.3a) [70]. The aligned reads were filtered to those with mapping quality ≥ 20 using samtools (1.9) [71]. Reads mapping to each gene were counted using featureCounts (1.6.3) for GENCODE (v28) genes [72]. Gene counts were converted to Fragments Per Kilobase Per Million (FPKM) using DESeq2 (1.22.2) [73]. Finally, the FPKM matrix was quantile normalized using the preprocessCore (1.44) package and z-score transformed.

### Estimating DNA allelic imbalance using exome-seq

We realigned exome-seq reads from tumor and normal samples to the reference genome (hg19) using BWA-ALN and filtered the sequencing reads to remove mapping bias using WASP [74]. We obtained allele-specific read counts at heterozygous sites (excluding multiallelic sites) for normal tissues using the *CollectAllelicCounts* from GATK (4.1.1). We assumed that most heterozygous sites in normal tissues are germline polymorphisms and obtained allele-specific read counts at the shared positions for matched tumor samples. We analyzed shared heterozygous positions because this facilitates direct comparison of reference allele proportions between tumor and paired normal tissues.

To model DNA allelic imbalance over large genomic segments, we sorted exons by their genomic coordinates and grouped 20 consecutive exons into genomic bins. Next, we assigned the heterozygous sites which were covered by at least 10 reads to the genomic bins. To ensure robust regional DNA allele imbalance estimates, we retained genomic bins with at least 10 heterozygous sites for DNA allele imbalance analysis.

To model allele-specific read counts. we assumed that the reference allele count at heterozygous sites is beta-binomially distributed. The likelihood for the allelic imbalance parameter, *a*, and the dispersion parameter, *d*, at a single heterozygous site *i* is then defined by:

$${\mathcal{L}}_i\left(a,d|{x}_{R,i},{n}_i\right)={p}_X\left(0.5+a,d\right)=\mathit{\Pr}\left(X={x}_{R,i}|p=0.5+a,d,n={n}_i\right)=\frac{B\left({x}_{R,i}+d\left(0.5+a\right),{n}_i-{x}_{R,i}+d\left(0.5-a\right)\right)\left(\genfrac{}{}{0pt}{}{n}{x_{R,i}}\right)}{B\left(d\left(0.5+a\right),d\left(0.5-a\right)\right)}$$

where *p*_*X*_() is the beta binomial probability mass function; *x*_*R*, *i*_ is the observed reference allele count from overlapping reads at site *i*; *n*_*i*_ is the total count of overlapping reads matching the reference or alternate allele at site *i*; *p* is the reference allele proportion; *a* is the allelic imbalance parameter and is defined over the range [−0.5,0.5]; *d* is the beta binomial dispersion parameter; and *B*() is the beta function. To perform likelihood calculations, we used the beta-binomial probability mass function (dbetabinom) provided by the rmutil (1.1.5) package, which is described in the vignette (https://cran.r-project.org/web/packages/rmutil/rmutil.pdf).

We estimate a single-dispersion parameter across all heterozygous sites genome-wide. This is accomplished by fixing *a* to 0 and finding the value of *d* that maximizes the total likelihood across all heterozygous sites in either a normal or a tumor sample:

$$\hat{d}={\mathrm{argmax}}_d{\prod}_i{\mathcal{L}}_i\left(a=0,d|{x}_{R,i},{n}_i\right)$$

After estimating the dispersion parameter, we estimate the allelic imbalance and compute the likelihood for bins. In the case of DNA allelic imbalance, a bin consists of 20 consecutive exons as described above. In the case of RNA allelic imbalance, we utilize the set of heterozygous sites within the exons of the gene being considered as described below. To estimate the imbalance of a bin, we combine information across all of the heterozygous sites within the bin. Since we do not know the phasing of the alleles, we consider all possible phasings (i.e., haplotype configurations), when computing the likelihood. Under the assumption that all phasings are equally probable, the likelihood of the parameters for bin *B* is:

$${\mathcal{L}}_B\left(a,d=\hat{d}|{x}_R,n\right)=\frac{1}{\left\Vert {H}_B\right\Vert}\prod_{h\in {H}_B}\prod_{i\in B\ }\left(1-{h}_i\right)\left({\mathcal{L}}_i\left(a,d=\hat{d}|{x}_{R,i},{n}_i\right)\right)+\left({h}_i\right)\left({\mathcal{L}}_i\left(a,d=\hat{d}|{n}_i-{x}_{R,i},{n}_i\right)\right)$$

where *H*_*B*_ is the set of all possible phasings (i.e., hapolotype configurations) for bin *B*. A phasing is defined by a vector of 0s and 1s, with each element corresponding to a heterozygous site. Element *i* of the phase vector is set to *h*_*i*_ = 0 if the reference allele for site *i* is on “chromosome A” and set to *h*_*i*_ = 1 if the reference allele for site *i* is on “chromosome B”. “Chromosome A” is defined as the chromosome that carries the reference allele for the first heterozygous site in the bin. In total there are ‖*H*_*B*_‖ = 2^*m* − 1^ possible phasings, where *m* is the number of heterozygous sites within the bin. While the number of phasings grows exponentially with *m*, the number of heterozygous sites in the bin, the likelihood for the bin can be computed efficiently in linear time using dynamic programming:

$$T\left[1\right]\leftarrow {\mathcal{L}}_1\left(a,d=\hat{d}|{x}_{R,1},{n}_1\right)$$

$$for\ i\ \mathrm{in}\ 2,\dots, m\ do$$

$$T\left[i\right]\leftarrow \left(T\left[i-1\right]\right)\ \left({\mathcal{L}}_i\left(a,d=\hat{d}|{x}_{R,i},{n}_i\right)+{\mathcal{L}}_i\left(a,d=\hat{d}|{n}_i-{x}_{R,i},{n}_i\right)\right)$$

$$return\ \frac{T\left[m\right]}{\left\Vert {H}_B\right\Vert }$$

where *T* is an array of length *m* that is used to compute the cumulative likelihood.

We performed one dimensional optimization to obtain maximum likelihood estimates of *a* for each bin. We perform this procedure separately for tumor and normal samples to obtain estimates of *a* for tumor (*a*_*tumor*_) and normal samples (*a*_*normal*_). We then calculate the difference in *a* between tumor and normal samples,*δ*_*a*_ as follows:

$${\delta}_a=\left|{a}_{tumor}\right|-\left|{a}_{normal}\right|$$

Finally, to create contiguous segments of allelic imbalance, we performed Circular Binary Segmentation (CBS) on *δ*_*a*_ using the DNAcopy package (1.56.0) [39]. We defined the aggregate value for *δ*_*a*_ obtained from CBS as the “SCNA score”. Plots for *δ*_*a*_ and SCNA scores were generated using Gviz (1.26.5), ComplexHeatmap (1.20.0), and gplots (3.0.1.1) [75].

### Estimating allele-specific expression per gene using RNA-seq reads

We filtered RNA-seq reads for mapping bias and obtained allele specific read counts at heterozygous positions using WASP [74]. WASP uses random sampling to ensure that allele counts at nearby heterozygous sites are independent. Specifically, when a read overlaps multiple sites, the allelic count is incremented at only one of the sites, which is selected randomly. We observed that at most heterozygous sites overlapping RNA-seq reads only match the reference or alternate alleles. However, some sites also have reads that match neither allele, which we refer to as “other” reads. “Other” reads may reflect sequencing errors or mis-mapped reads so, prior to ASE analysis, we removed all heterozygous sites where the “other” read count was greater than two.

Genotyping errors can create a false signal of allelic imbalance. For the SCNA analysis above, DNA imbalance is estimated from many heterozygous sites within each bin and then estimates from multiple bins are combined with circular binary segmentation. Since many heterozygous sites are used for this analysis, genotyping errors can be ignored without a major effect. However, when we calculate ASE for a gene, we only consider heterozygous sites within the exons of the gene. Thus, when estimating ASE for a single gene, we often utilize a small number of heterozygous sites, so it is desirable to account for genotyping errors. To control for genotyping errors, we calculate the genotyping error rate, *ϵ*_*G*, *i*_, for each heterozygous site *i* directly from the genotype quality (GQ) scores provided by GATK:

$${\epsilon}_{G,i}={10}^{-\frac{G{Q}_i}{10}}$$

When a genotyping error occurs, a heterozygous site can be homozygous reference (0/0) or alternate (1/1), and we assume these two possibilities are equally likely. When there is a genotyping error and the sample is homozygous, all sequencing reads that come from one of the two alleles must arise due to sequencing or mapping errors. We use the parameter *ϵ*_*S*_ to describe the frequency of these sequencing errors. The likelihood of the parameters for a single heterozygous site is then:

$${\mathcal{L}}_i\left({a}_i,d,{\epsilon}_S|{x}_{R,i},{n}_i,{\epsilon}_{G,i}\right)=\left(1-{\epsilon}_{G,i}\right){p}_X\left(0.5+a,d\right)+{\epsilon}_{G,i}\left(0.5\ {p}_X\left({\epsilon}_S,d\right)+0.5\ {p}_X\left(1-{\epsilon}_S,d\right)\right)=\left(1-{\epsilon}_{G,i}\right)\mathit{\Pr}\left(X={x}_{R,i}|p=0.5+a,d=d,n={n}_i\right)+\left(0.5{\epsilon}_{G,i}\right)\mathit{\Pr}\left(X={x}_{R,i}|p={\epsilon}_s,d=d,n={n}_i\right)+\left(0.5{\epsilon}_{G,i}\right)\mathit{\Pr}\left(X={x}_{R,i}|p=1-{\epsilon}_S,d=d,n={n}_i\right)$$

where, as described above, *p*_*X*_() is the probability mass function for the beta binomial distribution.

To find the maximum likelihood estimate of *d* and *ϵ*_*S*_, we fix *a* to 0 and optimize *d* and *ϵ*_*S*_ over all heterozygous sites overlapping exons:

$$\hat{d},\hat{\epsilon_S}={\mathrm{argmax}}_{d,{\epsilon}_s}{\prod}_i{\mathcal{L}}_i\left({a}_i,d,{\epsilon}_S|{x}_{R,i},{n}_i,{\epsilon}_{G,i}\right)$$

For optimization, we used the L-BFGS-B algorithm implemented by the “*optim”* function provided by the stats package in R-4.0.1.

To estimate the ASE of a gene within a sample, we obtain a maximum likelihood estimate of *a*, keeping the dispersion and sequencing error rate fixed to their genome-wide estimates ($\hat{d}$ and $\hat{\epsilon_S}$). We combine information across all heterozygous sites within each gene. To combine information across heterozygous sites, we use the same approach described in the DNA imbalance section above. We group all of heterozygous sites that fall within the exons of a gene into a “bin” and compute likelihoods that consider all possible phasings of alleles. We use a likelihood ratio test to compare the alternative model (with a free *a* parameter) to the null model of no allelic imbalance (with *a* fixed to *a* = 0) and to compute *p*-values. We correct the *p*-values for multiple testing using the Benjamini-Hochberg method. To make it clear when we are referring to allelic imbalance in RNA instead of DNA, we refer to *a* for RNA-seq read as *a*_*RNA*_.

### Gene Ontology enrichment analysis

Gene Ontology (GO) enrichment analysis for Biological Processes (BP) was performed using topGO (2.34.0). Enrichment was calculated using Fisher’s exact test, and all genes tested for ASE in neuroblastoma were used as the universe.

### Correlation between DNA allele-imbalance and SNP-array predictions

We used GenomicRanges (1.34.0) to find overlaps between SCNAs detected using our DNA allelic-imbalance method and SCNA predictions obtained from TARGET which were generated using HumanHap 550K Beadchip (SNP-array) [41]. For segments which show at least a 50% overlap, we computed Spearman’s correlation between segmented DNA allele imbalance (i.e., SCNA score) and corrected Log R ratio estimated using SNP-array data from TARGET [41].

### Association between allele-specific expression and SCNAs

We assigned our candidate genes to genomic segments predicted to be SCNAs based on the location of their promoters (transcription start site +/− 1500bp) using GenomicRanges (1.34.0) [76] and computed Spearman’s correlation between ASE (*a*_*RNA*_) and SCNA score. We corrected the *p*-values for multiple testing using the Benjamini-Hochberg procedure. We only tested genes with non-zero variance in both SCNA scores and *a*_*RNA*_ and with an SCNA score in at least one sample ≥ 0.09. Manhattan plots for Spearman’s correlation coefficient were generated using ggbio (1.30.0) [77].

### Correlations between allele-specific expression and promoter methylation

In Human Methylation 450K BeadChIP array (HM450K) data, the ratios of intensities between methylated and unmethylated CpG probes are referred to as beta values (β) and range from 0 (unmethylated) to 1 (completely methylated). We downloaded a pre-computed β matrix for 87 neuroblastoma samples from TARGET and annotated the CpG probe positions based on GENCODE (version 28) genes. Then, we computed the mean β for promoter regions (transcription start site +/− 1500 bp) and computed Spearman’s correlations between promoter methylation and ASE (a_RNA_) for 1043 NB-ASE genes. We corrected the *p*-values for multiple testing using the Benjamini Hochberg procedure. We only tested genes with at least 3 CpG probes within promoter regions.

### Survival and expression analysis in neuroblastoma patients

We analyzed the SEQC/MAQC-III Consortium dataset consisting of 498 individuals using the R2: Genomics Analysis and Visualization Platform (http://r2.amc.nl) to generate Kaplan-Meier survival plots for neuroblastoma [60]. We also downloaded a normalized gene expression matrix (i.e., log (1 + FPKM)) for SEQC/MAQC-III Consortium dataset from Gene Expression Omnibus (GSE49711) [60] and generated gene expression heatmaps using ComplexHeatmap (1.20.0) and ggplot2 (3.2.1) [75, 78].

### Cell culture and transfection

The SK-N-BE(2) and SK-N-SH cell lines were purchased from the American Type Culture Collection (www.atcc.org) and grown in a humidified chamber with 5% CO_2_ in RPMI 1640 medium (Gibco, #11875119) supplemented with 10% fetal bovine serum (FBS), 2mM l-glutamine, sodium pyruvate, non-essential amino acids, and 1% antibiotic antimycotic.

For stable transfection, the cell lines were seeded in 6-well plates and allowed to grow to 70% confluence. Cells were transfected with shRNAs purchased from Sigma-Aldrich (shPTPRH-373, #TRCN0000355579; shPTPRH-1136, #TRCN0000355581; shPTPRH-1947, #TRCN0000355580; shPTPRH-3265, TRCN0000355631; shPTPRH-3621, #TRCN0000002866) with jetOPTIMUS® DNA transfection Reagent (VWR, #76299-632) following the protocol provided by the manufacturer. The cells were selected in RPMI containing puromycin 24hrs after transfection (SK-N-BE(2), 1μg/ml puromycin and SK-N-SH, and 1.25μg/ml puromycin). The stably transfected cells were maintained in complete medium supplemented with puromycin until use.

### Quantitative RT-PCR analysis

SK-N-BE(2) and SK-N-SH cells were seeded in 6-well plates and allowed to grow to 50% confluence. RNA was isolated from the cells with Zymo Quick-RNA Miniprep kit (VWR, #76299-632) according to the manufacturer’s instructions. Total RNA was reversed transcribed into cDNA using High-Capacity cDNA Reverse Transcription Kit (Fisher Scientific, #4374966). Real-time PCR was performed using iTaq™ Universal SYBR Green Supermix (Bio-Rad Laboratories, #1725122) on a Bio-Rad CFX96 system. Gene expression was analyzed by the log2ΔΔCt method.

### Immunoblotting

SK-N-BE(2) and SK-N-SH cells were seeded in 6-well plates and allowed to grow to 70–80% confluence. Cells were lysed in RIPA buffer, and the protein concentration was determined by a Pierce BCA protein assay (Life Technologies, #23225). Equal amounts of protein were loaded into 8% Bolt™ Bis-Tris Plus gels (Life Technologies, #NW00085BOX), separated by SDS-PAGE and then transferred to PVDF membranes. The membranes were incubated with primary antibodies (PTPRH antibody, 1:1000, Fisher Scientific, #PIPA531340) overnight at 4^o^C. The membranes were then probed with appropriate horseradish peroxidase-conjugated secondary antibodies (Goat Anti-Rabbit IgG(H+L)-HRP Conjugate, Bio-rad Laboratories, #170-6515). The immunoblots were visualized with SuperSignal West Pico Plus Chemiluminescent Substrate (Life Technologies, #PI34580).

### Proliferation and migration assays

SK-N-BE(2) and SK-N-SH cells were plated in 96-well plates at a seeding density of 7500 cells/well and allowed to attach overnight. Cells were then monitored by continuous live-cell imaging in the IncuCyte® Zoom^TM^ system (Essen Bioscience), and 10x phase contrast images were taken every 3h. Cell confluence in each image was calculated by the IncuCyte® analysis software.

For migration assays, SK-N-BE(2) and SK-N-SH cells were seeded in IncuCyte® Imagelock 96-well plates (Essen Bioscience, #4379) at seeding densities between 100,000 and 200,000 cells/well and allowed to grow to 100% confluence. Cells were treated with 10μM cytosine arabinoside (Sigma-Aldrich, #C1768) for 4h, and then, identical scratch wounds were made in each well with a 96-pin WoundMaker (Essen Bioscience). Wound closure was monitored by continuous live-cell imaging in the IncuCyte Zoom^TM^ (Essen Bioscience), and 10x phase contrast images were taken every 6h. The wound closure percentage was calculated using IncuCyte Scratch Wound Analysis Software.

To analyze the cellular proliferation and migration rates, we computed the mean confluence and mean wound closure at each time point across replicates. To allow for non-linearity in both types of data, we performed cubic spline regression with 3 knots (two degrees of freedom) to describe the change in confluence or wound closure with time. To quantify differences in proliferation or migration between knockdown and parental cells, we included an interaction term between knockdown status and the time spline in the model. Specifically, we utilized the following linear model command in R, where “response” is the confluence or wound closure, “ns” is the cubic spline function, and “cond” is a factor giving knockdown status (parental or knockdown):

$$\mathrm{lm}\left(\mathrm{response}\sim \mathrm{cond}\ast \mathrm{ns}\left(\mathrm{time},\mathrm{df}=2\right)\right)$$

Significance of the interaction term was determined with an F-test.

## Supplementary Information

**Additional file 1: Fig. S1.** Validation of SCNA scores using SNP-array data. A SCNA predictions based on DNA allelic imbalance compared to SNP-array predictions. Left panel shows SCNA scores across chromosome 1 estimated from DNA allelic imbalance from 33 neuroblastoma patients. Right Panel shows Corrected Log R ratio (or Corrected LRR) calculated from SNP array data for the same 33 patients. Corrected LRR is defined as aneuploidy corrected total probe intensity of a given genomic segment relative to a canonical set of normal controls and directly available from the TARGET. B Spearman’s rank correlation between SCNA score and absolute Corrected LRR for chromosome 1 (Spearman’s correlation coefficient = 0.614, *p*-value = 3.51e-06). Exome-seq and SNP arrays use different sets of SNPs to predict SCNAs. Therefore, the Circular Binary Segmentation (CBS) algorithm tends to output segments which do not share the same genomic start and end positions. To directly compare SCNA detection using DNA allele imbalance and SNP array, we first calculated the fraction overlap between genome segments identified by the respective methods. Next, we performed pairwise Spearman’s correlation between SCNA score and absolute Corrected LRR for genomic segments with fraction of overlap ≥ 0.5 or 50%. The points in the correlation scatter plot are colored by fraction overlap. The two points labelled PANRVJ correspond to two disjointed SCNAs spanning chr1:1922327-9171333 and chr1:49201909-120298048. These regions showed absolute Corrected LRR < 0.5 and were annotated as copy neutral by SNP array. We suspect that these segments may be copy-neutral loss of heterozygosity regions, which are not detectable using direct analysis of SNP-arrays in TARGET. **Fig. S2.** SCNA predictions for chromosome 11. A Comparison between DNA-imbalance SCNA predictions and CNVkit predictions for chr11. Left panel shows heatmap of δ" for 96 neuroblastoma patients. Right panel shows fold-change in normalized read coverage between tumor and normal tissues estimated using CNVkit. B Comparison between DNA-imbalance predictions and SNP-array predictions for chr11. Left panel shows SCNA score across 33 neuroblastoma patients with SNP-array data in TARGET. Right panel shows corrected LRR calculated array SNP-array available through TARGET. C Spearman’s rank correlation between SCNA score and absolute corrected LRR for chr11 (Spearman’s correlation coefficient = 0.565, *p*-value = 2.3e-05). The points are colored based on fraction of overlap between genomic regions detected by our method and genomic regions from SNP-array based predictions. D Spearman’s rank correlation between ASE (aRNA) and SCNA score for *MTMR2*, a gene located within a common deletion segment on cytoband 11q21 (Spearman’s correlation coefficient = 0.64, *p*-value = 0.0001). **Fig. S3.** Detection of rare SCNAs on chromosome 16. A Left panel shows the heatmap of δ" for chromosome 16. Right panel shows the log2 fold-change in normalized read coverage between tumor and normal tissues estimated using CNVkit for chromosome 16. B Comparison between SCNA score and SNP-array predictions (i.e. corrected LRR) for chromosome 16 for 33 neuroblastoma samples. C Spearman’s rank correlation between SCNA score and absolute corrected LRR for chromosome 16 (Spearman’s correlation coefficient=0.59, *p*-value = 5.04e-05) for overlapping genomic regions. The points are colored based on fraction overlap between genomic regions detected by our method and genomic regions from SNP-array based predictions. D Spearman’s rank correlation between ASE (aRNA) and SCNA score for *CDT1*, a gene located in the distal region of the q-arm (i.e., 16q24.3) (Spearman’s correlation coefficient = 0.58, FDR corrected *p*-value = 2e-06). **Fig. S4.** Haplo-insufficient tumor suppressors within common SCNAs may be dysregulated by secondary mechanisms. Spearman’s correlation between ASE (aRNA) and SCNA score for example chromosome 1p and chromosome 11q deletion genes: A *CHD5*, B *UBE4B*, C *CADM1*, and D *ATM*. Several samples show ASE in the absence of SCNAs. **Fig. S5.** Allele-specific expression, gene expression, promoter methylation, and survival for *TFAP2B*. A ASE (*a*RNA) of *TFAP2B* in neuroblastoma and adrenal gland tissues. B Reference and alternate allele proportion for RNA-seq and exome-seq reads at heterozygous sites which were used to estimate ASE for *TFAP2B*. C Correlation between ASE (*a*RNA) and promoter methylation for *TFAP2B*. DNA methylation data was missing for 1 neuroblastoma sample (PAMVRA). The two samples with significant ASE are among those with the greatest promoter methylation. ASE of *TFAP2B* is correlated with its promoter methylation, however this correlation is not significant under an FDR threshold of 10% (Spearman’s rho= 0.604, FDR corrected *p*-value = 0.116). D Spearman’s correlation between ASE (*a*RNA) and gene expression for *TFAP2B.* E Genomic distribution of HM450K β-values for *TFAP2B* locus. The *TFAP2B* promoter is highlighted (gold box). F Expression profile of *TFAP2B* across different stages of disease for 498 neuroblastoma patients obtained from SEQC/MAQC-III Consortium data set. We observed loss of expression of *TFAP2B* in stage 4 or metastatic disease suggesting this gene might act as a tumor suppressor. G Kaplan Meier survival analysis for *MYCN* nonamplified patients from the SEQC/MAQC-III Consortium data set. **Fig. S6.** Quantile-quantile plot for Spearman’s correlation analysis between ASE (aRNA) and promoter methylation for 1,043 NB-ASE genes. Under an FDR of 10% only the expression of *ODZ4* is significantly correlated with promoter methylation. **Fig. S7.** Knockdown of PTPRH in neuroblastoma cell lines. A, B *PTPRH* expression measured by qPCR in (A) SK-N-SH or (B) SK-N-BE(2) neuroblastoma cell lines stably transfected with 5 different shRNAs targeting *PTPRH*. Gene expression is plotted as 2-DDCt normalized to *HPRT1* expression. C, D Western blot of PTPRH and GAPDH protein expression for the same (C) SK-N-SH and (D) SK-N-BE(2) cell lines. shRNA shPTPRH-373 consistently reduced gene and protein expression of *PTPRH* in both SK-N-SH and SK-N-BE(2) cells and was used for all downstream experiments. E, F Uncropped versions of the western blots of PTPRH and GAPDH protein expression in (E) SK-N-SH and CHP212 cells, and (F) SK-N-BE(2) cells. Note that we only used SK-NSH and SK-N-BE(2) cells for the proliferation and wound healing assays shown in main text Fig. 6.

**Additional file 2: Table S1.** Results from Allele-Specific Expression (ASE) analysis of RNA-seq data for 96 neuroblastoma samples from TARGET. **Table S2.** Number of significant (FDR ≤ 0.1 or 10%) and testable (i.e., at least 1 heterozygous site with ≥ 10 reads) samples for neuroblastoma tumors, adrenal gland, and whole-blood tissues for all significant ASE genes. Genes were considered to have neuroblastoma-specific ASE if they met these criteria; a) testable in ≥ 10 neuroblastoma and normal (i.e., adrenal-gland and whole-blood) samples and b) significant in ≥3 neuroblastoma and significant ≤ 1 normal sample. The prefix “r.” indicates the number of samples showing significant ASE and “N.” indicates the number of samples testable for ASE. **Table S3.** Top 20 Gene Ontology (Biological processes) categories enriched for 1,043 NB-ASE genes. **Table S4.** SCNA scores for 96 neuroblastoma patient samples. **Table S5.** Spearman’s correlation between ASE (a_RNA_) for 1,043 NB-ASE genes and SCNA score for overlapping genomic segments. **Table S6.** Table of high-impact somatic mutations mapping to NB-ASE genes detected using exome-seq data using Variant Effect Predictor (VEP) in 96 neuroblastoma tumors. **Table S7.** Spearman’s correlation between ASE (aRNA) and mean promoter methylation for 1,043 NB-ASE genes. **Table S8.** Spearman’s correlation between ASE (a_RNA_) and gene expression (z-score normalized Fragments per Kilobase Per Million mapped) for 1,043 NB-ASE genes.

**Additional file 3.** Review history.

## Data Availability

The datasets analyzed in this study are available in the following repositories. Data from the Genotype-Tissue Expression (GTEx) Project are available from the Database of Genotypes and Phenotypes (dbGAP) via accession phs000424.v7.p2 (https://www.ncbi.nlm.nih.gov/projects/gap/cgi-bin/study.cgi?study_id=phs000424.v7.p2) [79]. Data from the Therapeutically Applicable Research to Generate Effective Treatments (TARGET) initiative are available from dbGAP via accession phs000218.v1.p1 (https://www.ncbi.nlm.nih.gov/projects/gap/cgi-bin/study.cgi?study_id=phs000218.v1.p1) [7]. Gene expression data from the SEQC/MAQC-III Consortium are available from the Gene Expression Omnibus (GEO) via accession GSE49711 (https://www.ncbi.nlm.nih.gov/geo/query/acc.cgi?acc=GSE49711) [80]. Survival data for this dataset are available from the R2: Genomics Analysis and Visualization Platform (http://r2.amc.nl). Source code for RNA and DNA allelic imbalance analyses are available from GitHub at https://github.com/Arkosen/Allele-imbalance-analysis-of-RNA-and-DNA and in archived form at 10.5281/zenodo.6229172.
