# Supplementary material for: Allele-specific expression reveals genes with recurrent cis-regulatory alterations in high-risk neuroblastoma
Source: Genome Biol. 2022 Mar 4;23:71. doi: 10.1186/s13059-022-02640-y (PMC8896304; doi:10.1186/s13059-022-02640-y)
Supplement: Supplementary file 1 — Additional file 1: Fig. S1. Validation of SCNA scores using SNP-array data. A SCNA predictions based on DNA allelic imbalance compared to SNP-array predictions. Left panel shows SCNA scores across chromosome 1 estimated from DNA allelic imbalance from 33 neuroblastoma patients. Right Panel shows Corrected Log R ratio (or Corrected LRR) calculated from SNP array data for the same 33 patients. Corrected LRR is defined as aneuploidy corrected total probe intensity of a given genomic segment relative to a canonical set of normal controls and directly available from the TARGET. B Spearman’s rank correlation between SCNA score and absolute Corrected LRR for chromosome 1 (Spearman’s correlation coefficient = 0.614, p-value = 3.51e-06). Exome-seq and SNP arrays use different sets of SNPs to predict SCNAs. Therefore, the Circular Binary Segmentation (CBS) algorithm tends to output segments which do not share the same genomic start and end positions. To directly compare SCNA detection using DNA allele imbalance and SNP array, we first calculated the fraction overlap between genome segments identified by the respective methods. Next, we performed pairwise Spearman’s correlation between SCNA score and absolute Corrected LRR for genomic segments with fraction of overlap ≥ 0.5 or 50%. The points in the correlation scatter plot are colored by fraction overlap. The two points labelled PANRVJ correspond to two disjointed SCNAs spanning chr1:1922327-9171333 and chr1:49201909-120298048. These regions showed absolute Corrected LRR < 0.5 and were annotated as copy neutral by SNP array. We suspect that these segments may be copy-neutral loss of heterozygosity regions, which are not detectable using direct analysis of SNP-arrays in TARGET. Fig. S2. SCNA predictions for chromosome 11. A Comparison between DNA-imbalance SCNA predictions and CNVkit predictions for chr11. Left panel shows heatmap of δ" for 96 neuroblastoma patients. Right panel shows fold-change in normalized read coverage be [file 13059_2022_2640_MOESM1_ESM.pdf]

Additional file 1: Figures S1-S7

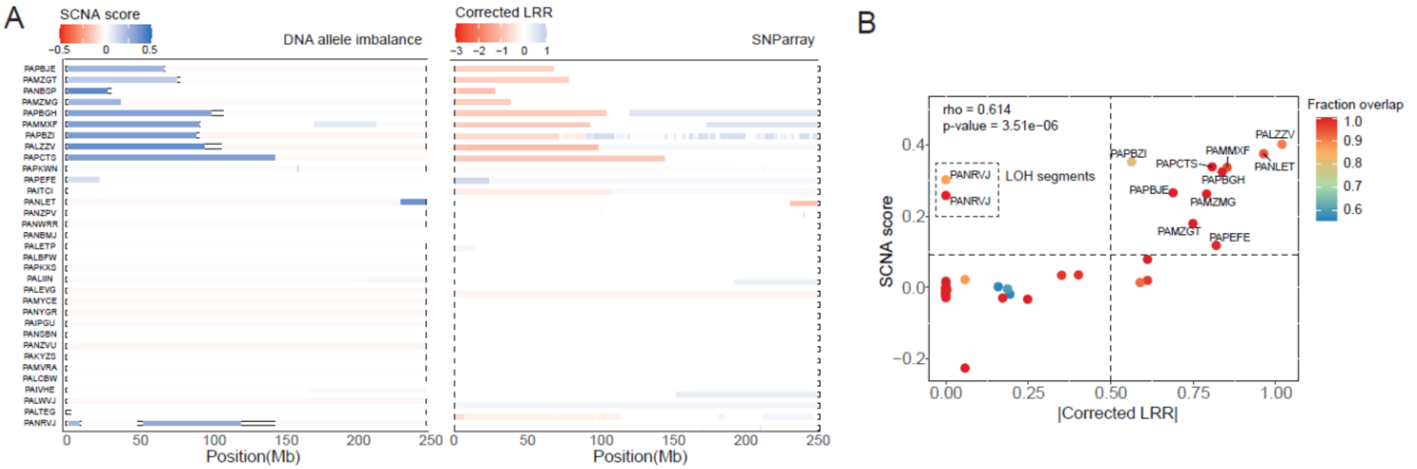

Fig. S1: Validation of SCNA scores using SNP-array data. **A** SCNA predictions based on DNA allelic imbalance compared to SNP-array predictions. Left panel shows SCNA scores across chromosome 1 estimated from DNA allelic imbalance from 33 neuroblastoma patients. Right Panel shows Corrected Log R ratio (or Corrected LRR) calculated from SNP array data for the same 33 patients. Corrected LRR is defined as aneuploidy corrected total probe intensity of a given genomic segment relative to a canonical set of normal controls and directly available from the TARGET. **B** Spearman's rank correlation between SCNA score and absolute Corrected LRR for chromosome 1 (Spearman's correlation coefficient = 0.614, p-value = 3.51e-06). Exome-seq and SNP arrays use different sets of SNPs to predict SCNAs. Therefore, the Circular Binary Segmentation (CBS) algorithm tends to output segments which do not share the same genomic start and end positions. To directly compare SCNA detection using DNA allele imbalance and SNP array, we first calculated the fraction overlap between genome segments identified by the respective methods. Next, we performed pairwise Spearman's correlation between SCNA score and absolute Corrected LRR for genomic segments with fraction of overlap ≥ 0.5 or 50%. The points in the correlation scatter plot are colored by fraction overlap. The two points labelled PANRVJ correspond to two disjointed SCNAs spanning chr1:1922327-9171333 and chr1:49201909-120298048. These regions showed absolute Corrected LRR < 0.5 and were annotated as copy neutral by SNP array. We suspect that these segments may be copy-neutral loss of heterozygosity regions, which are not detectable using direct analysis of SNP-arrays in TARGET.

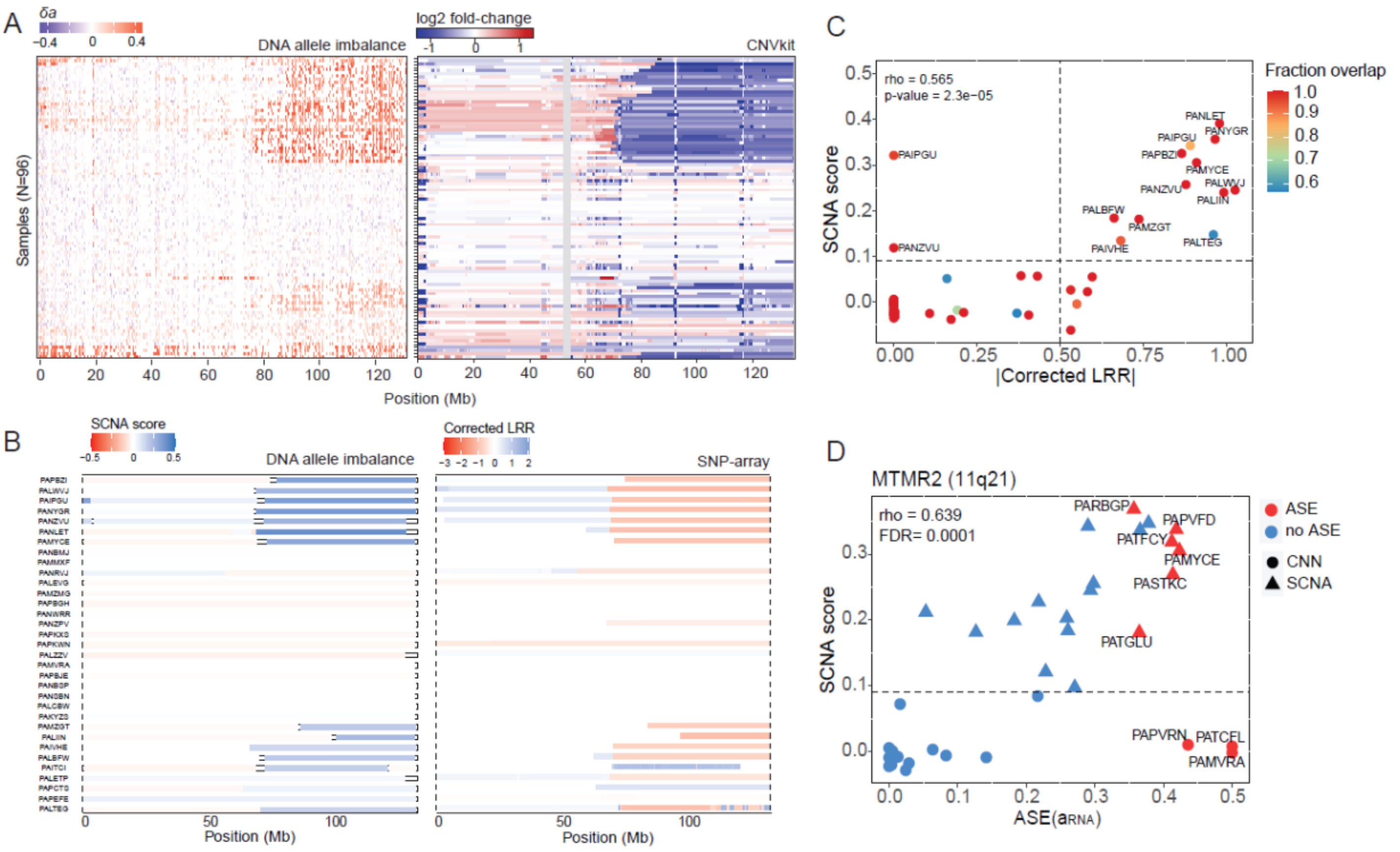

Fig. S2: SCNA predictions for chromosome 11. **A** Comparison between DNA-imbalance SCNA predictions and CNVkit predictions for chr11. Left panel shows heatmap of δ_a for 96 neuroblastoma patients. Right panel shows fold-change in normalized read coverage between tumor and normal tissues estimated using CNVkit. **B** Comparison between DNA-imbalance predictions and SNP-array predictions for chr11. Left panel shows SCNA score across 33 neuroblastoma patients with SNP-array data in TARGET. Right panel shows corrected LRR calculated array SNP-array available through TARGET. **C** Spearman's rank correlation between SCNA score and absolute corrected LRR for chr11 (Spearman's correlation coefficient = 0.565, p-value = 2.3e-05). The points are colored based on fraction of overlap between genomic regions detected by our method and genomic regions from SNP-array based predictions. **D** Spearman's rank correlation between ASE (a_{RNA}) and SCNA score for *MTMR2*, a gene located within a common deletion segment on cytoband 11q21 (Spearman's correlation coefficient = 0.64, p-value = 0.0001).

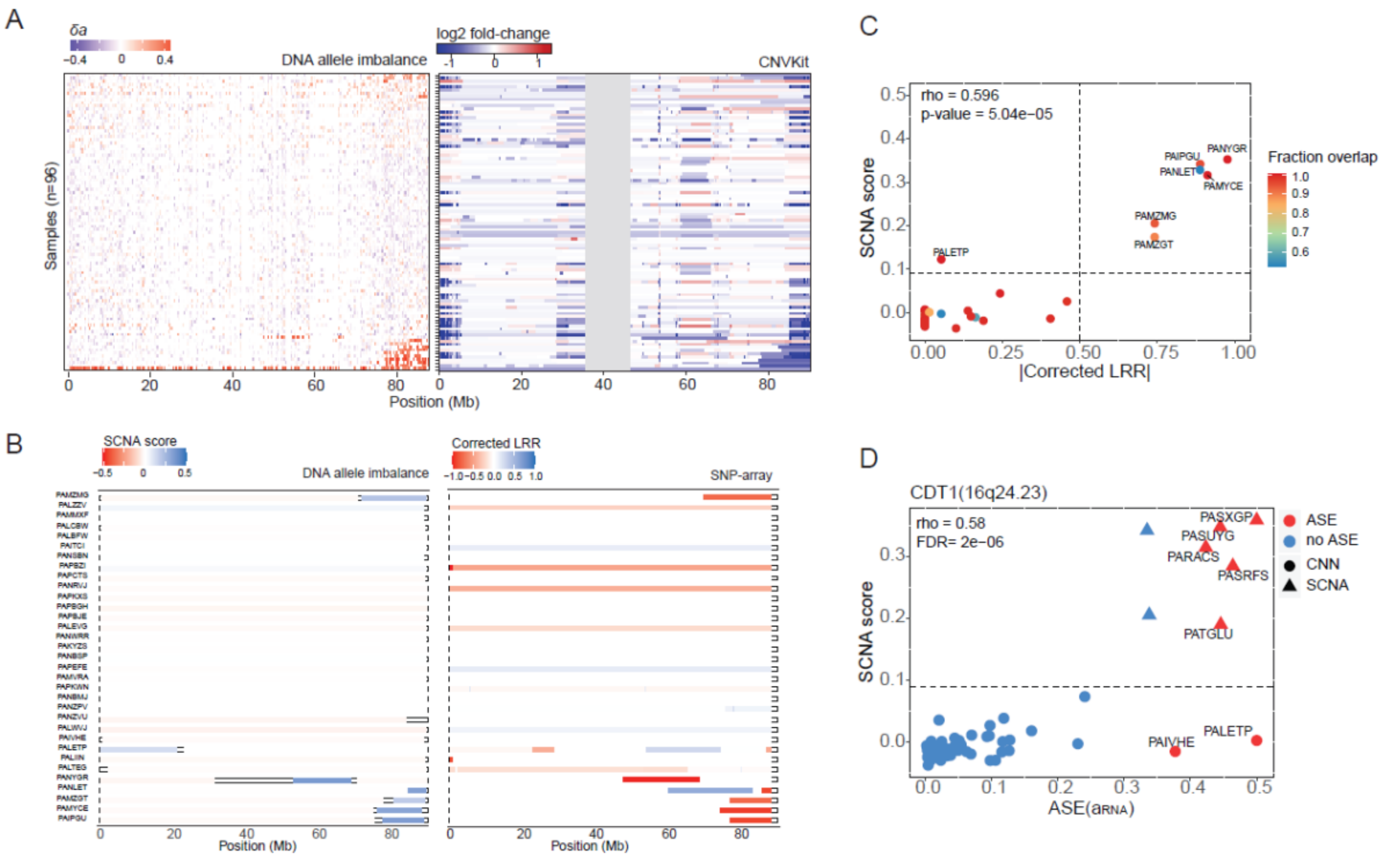

Fig. S3: Detection of rare SCNAs on chromosome 16. **A** Left panel shows the heatmap of δ_a for chromosome 16. Right panel shows the log2 fold-change in normalized read coverage between tumor and normal tissues estimated using CNVkit for chromosome 16. **B** Comparison between SCNA score and SNP-array predictions (i.e. corrected LRR) for chromosome 16 for 33 neuroblastoma samples. **C** Spearman's rank correlation between SCNA score and absolute corrected LRR for chromosome 16 (Spearman's correlation coefficient=0.59, p-value = 5.04e-05) for overlapping genomic regions. The points are colored based on fraction overlap between genomic regions detected by our method and genomic regions from SNP-array based predictions. **D** Spearman's rank correlation between ASE (a_{RNA}) and SCNA score for *CDT1*, a gene located in the distal region of the q-arm (i.e., 16q24.3) (Spearman's correlation coefficient = 0.58, FDR corrected p-value = 2e-08).

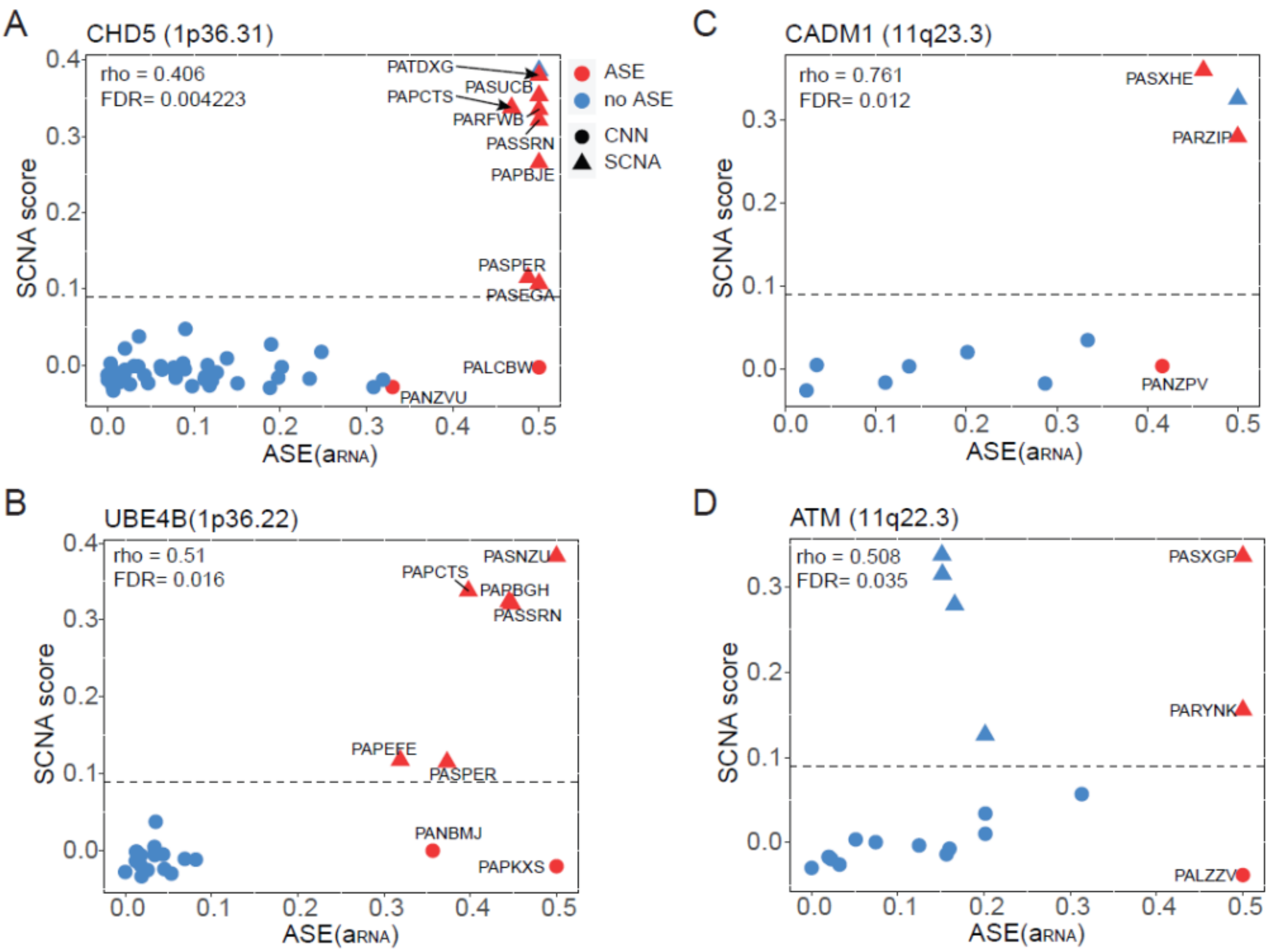

Fig. S4: Haplo-insufficient tumor suppressors within common SCNAs may be dysregulated by secondary mechanisms. Spearman's correlation between ASE (a_{RNA}) and SCNA score for example chromosome 1p and chromosome 11q deletion genes: **A** *CHD5*, **B** *UBE4B*, **C** *CADM1*, and **D** *ATM*. Several samples show ASE in the absence of SCNAs.

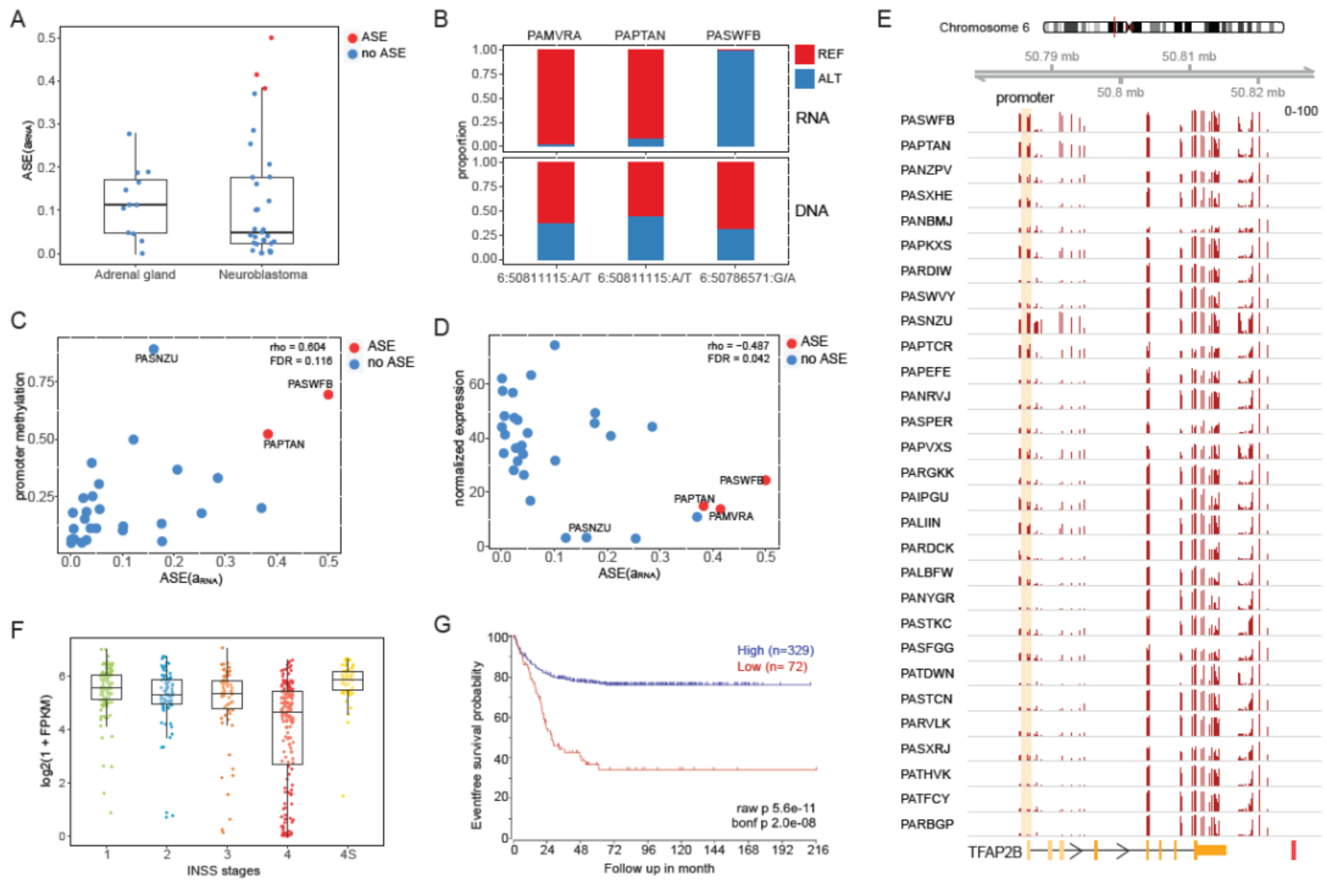

Fig. S5: Allele-specific expression, gene expression, promoter methylation, and survival for *TFAP2B*. **A** ASE (a_{RNA}) of *TFAP2B* in neuroblastoma and adrenal gland tissues. **B** Reference and alternate allele proportion for RNA-seq and exome-seq reads at heterozygous sites which were used to estimate ASE for *TFAP2B*. **C** Correlation between ASE (a_{RNA}) and promoter methylation for *TFAP2B*. DNA methylation data was missing for 1 neuroblastoma sample (PAMVRA). The two samples with significant ASE are among those with the greatest promoter methylation. Over all samples, ASE of *TFAP2B* is correlated with its promoter methylation, however this correlation is not significant under an FDR threshold of 10% (Spearman's rho= 0.604, FDR corrected p-value = 0.116). **D** Spearman's correlation between ASE (a_{RNA}) and gene expression for *TFAP2B. **E*** Genomic distribution of HM450K β-values for *TFAP2B* locus. The *TFAP2B* promoter is highlighted (gold box). **F** Expression profile of *TFAP2B* across different stages of disease for 498 neuroblastoma patients obtained from SEQC/MAQC-III Consortium data set. We observed loss of expression of *TFAP2B* in stage 4 or metastatic disease suggesting this gene might act as a tumor suppressor. **G** Kaplan Meier survival analysis for *MYCN* non-amplified patients from the SEQC/MAQC-III Consortium data set.

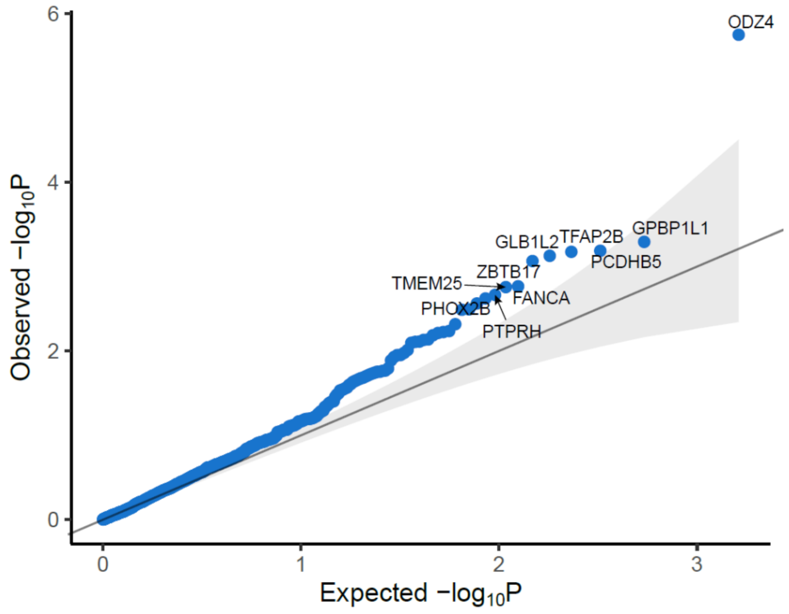

Fig. S6: Quantile-quantile plot for Spearman's correlation analysis between ASE (a_{RNA}) and promoter methylation for 1,043 NB-ASE genes. Under an FDR of 10% only the expression of *ODZ4* is significantly correlated with promoter methylation.

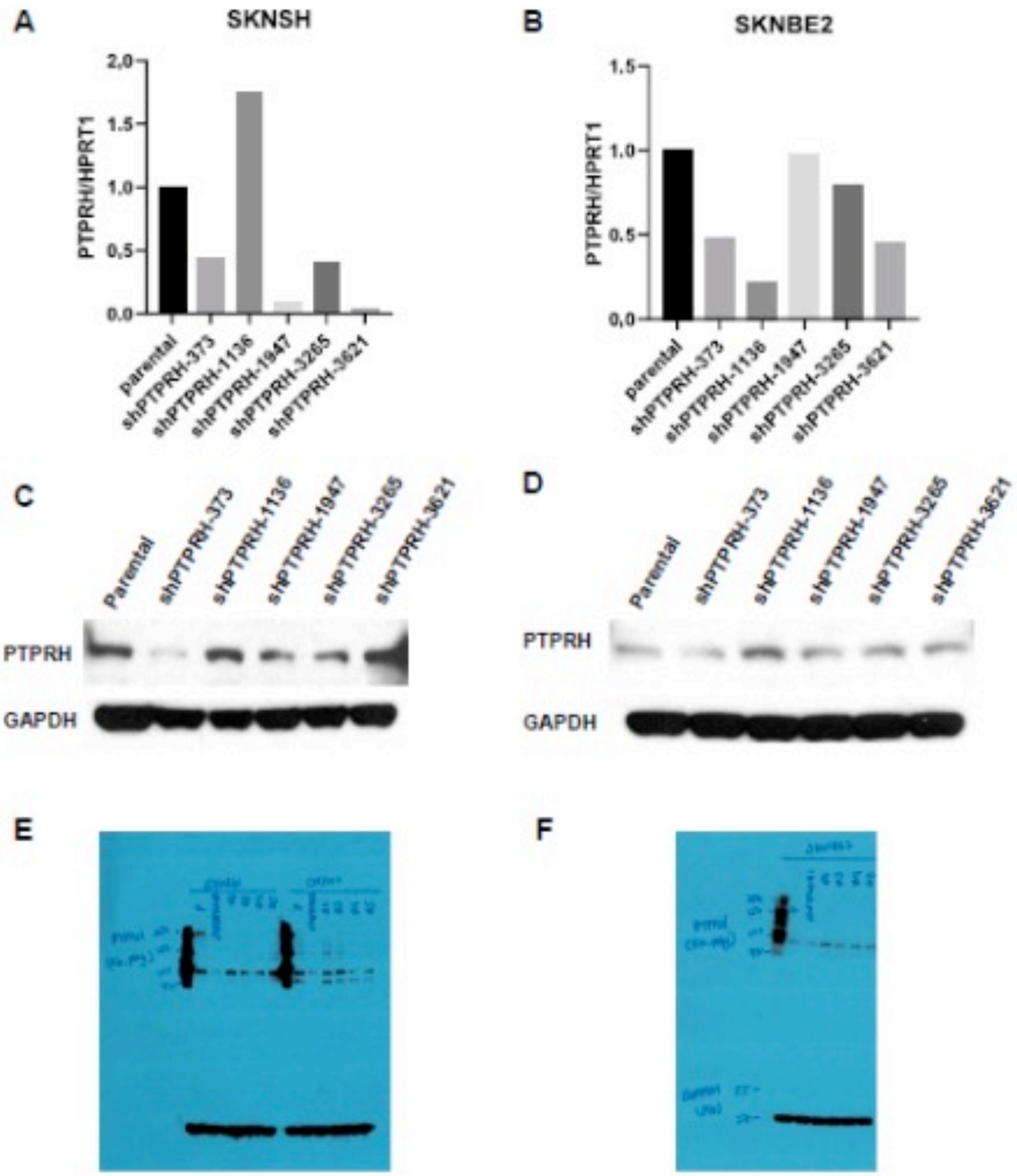

Fig. S7: Knockdown of PTPRH in neuroblastoma cell lines. **A, B** *PTPRH* expression measured by qPCR in (A) SK-N-SH or (B) SK-N-BE(2) neuroblastoma cell lines stably transfected with 5 different shRNAs targeting *PTPRH*. Gene expression is plotted as $2^{-\Delta\Delta Ct}$ normalized to *HPRT1* expression. **C**, **D** Western blot of PTPRH and GAPDH protein expression for the same (C) SK-N-SH and (D) SK-N-BE(2) cell lines. shRNA shPTPRH-373 consistently reduced gene and protein expression of *PTPRH* in both SK-N-SH and SK-N-BE(2) cells and was used for all downstream experiments. **E, F** Uncropped versions of the western blots of PTPRH and GAPDH protein expression in (E) SK-N-SH and CHP212 cells, and (F) SK-N-BE(2) cells. Note that we only used SK-N-SH and SK-N-BE(2) cells for the proliferation and wound healing assays shown in main text Fig. 6.
