# Supplementary material for: Allele-specific expression reveals genes with recurrent cis-regulatory alterations in high-risk neuroblastoma
Source: Genome Biol. 2022 Mar 4;23:71. doi: 10.1186/s13059-022-02640-y (PMC8896304; doi:10.1186/s13059-022-02640-y)
Supplement: Supplementary file 3 — Additional file 3. Review history. [file 13059_2022_2640_MOESM3_ESM.docx]

Review History

**First round of review**

**Reviewer 1**

**Are you able to assess all statistics in the manuscript, including the appropriateness of statistical tests used?**

Yes

**Comments to author:**

This is an interesting article that analyzes allele specific expression (ASE) in neuroblastoma, in order to find non-coding drivers of cancer. A nice aspect of the paper is that it considers allelic imbalance in the noncancerous setting, as estimated from GTEX expression data, in order to figure out when ASE observed in cancer samples is remarkable. Additionally, the paper gives an approach to detect copy number variation based on allelic imbalance in exome data. The authors find evidence that there are numerous genes with recurrent ASE in neuroblastoma, due either to copy number variation or to other, as yet undetected, alterations. The work showcases the power of ASE to highlight non protein coding driver events in cancer. Overall, the paper should be of an interest to a broad range of scientists working in cancer genomics, computational biology and neuroblastoma.

The authors should cite PMID 32078798, where Przytycki & Singh have shown that ASE can be used to detect driver genes in the breast cancer context- that work required matched normal RNA-seq data whereas the current manuscript uses GTEX data and additionally gives an approach to detect copy number variants.

The overall methodology given in the manuscript seems sound, but its write up needs to be improved substantially. More details are necessary in the section on "Estimating DNA allelic imbalance using exome-seq," and the notation there needs to be very carefully defined. Some examples: The likelihood does not seem to be written as a likelihood but as a probability. The authors should be more precise with notation (e.g., X_{R, i} is defined but X_{R.tumor, i} is not), and should clarify whether X_{R, i} refers to all reads (tumor and normal) mapping to that site. When P_{BB} is first used, the manuscript should state that this is the probability using beta binomial. It's not clear why the formula for L(X_{R, i}|parameters} is given considering both tumor and normal together, when ML estimates are likely done separately for tumor and normal. Please give more details on how dispersion parameter is estimated - Is this over sites in the bin? Are all sites assumed to be independent? What types of dispersion values are estimated? The formula in the middle of page 15 appears to be missing a multiplication sign.

The model assumes independence over sites, this may not be valid as nearby sites are correlated; the authors should comment on this.

For section on estimating allelic expression: What is n in the equation for epsilon_s? Please clarify where this comes from: "1/3rd of sequencing errors will be counted as alternate or reference alleles". What is little p? Where is episilon_g defined? Additional text that helps the reader understand the methodology is necessary.

**Reviewer 2**

**Are you able to assess all statistics in the manuscript, including the appropriateness of statistical tests used?**

Yes

**Comments to author:**

In this paper, Sen et al. analyzed allele-specific expression (ASE) in 96 high-risk neuroblastoma tumors to discover genes with cis-acting mutations that alter the dosage. The authors discovered that many genes exhibit ASE in copy neutral samples and are enriched for mutations that cause nonsense-mediated decay and 24 genes with reduced expression in stage 4 disease that have neuroblastoma-specific ASE that is independent of SCNAs. The authors generated some interesting hypotheses based on the ASE of genes, however, most of their hypotheses are not convincing. I have some technical questions and concerns about the proposed algorithm for ASE analysis and the in-vitro experiments. Blow, I list my specific comments:

Major comments:

1. Have the authors tried the haplotype phase algorithm in exom-seq data? As the proposed model was based on a 50% probability of SNPs on the same haplotype.

2. Were the authors being able to check the allele imbalance methylation on promoters of allelic imbalance genes?

3. In my opinion, it is a bit risky to keep SNP sites with sequence errors incorporated in the model as you assigned an equal probability of sequence errors to reference or alternative.

4. What was the way the authors used to call mutation in promoter regions and other non-coding regions with exom-seq?

5. I am not sure what are "ADRNLG" and "WHLBLD" in figures 1B and 1C?

6. On page 7 lines 27-29, the authors mentioned that 'We further examined the relationship between ASE and SCNAs for specific genes in the chromosome 1p and 3p deletion regions that are hypothesized to be tumor suppressors.', however, not sure why ASE and SCNAs for specific genes in chromosome 1p and 3p deletion regions are tumor suppressors, would the authors clarify more?

7. On page 7 line 47, the authors mentioned that 'We hypothesized that some ASE events are caused by nonsense-mediated decay (NMD) introduced by mutations that create premature stop codons including frameshifting indels, nonsense mutations, stop-loss mutations, or mutations that alter splicing', which is very interesting, however, there is no convincing evidence to support this hypothesis.

8. In Figure 6, it is unclear that if PTPRH knockdowns (KDs) in SK-N-BE (2) and SK-N-SH cells are allelic specific or not? If PTPRH knockdown is not in an allelic-specific manner, not sure how the in-vitro experiments validate the functions of ASE of PTPRH. The title of this paper is "Allele-specific expression links diverse cis-regulatory mutations to recurrent gene dysregulation in high-risk neuroblastoma", I am not sure how the ASE of PTPRH links to cis-regulatory mutations in high-risk neuroblastoma. There is no whole genome sequencing data in this paper, not sure how cis-regulatory mutations were identified. I would suggest the authors changing the title to better fit the content of this paper.

9. There is no in-vivo data to validate the function of PTPRH with ASE. It is not convincing if there is no in-vivo validation of PTPRH KDs, which is not difficult to perform.

10. On page 10 line 24, the authors mentioned that 'ASE of PTPRH is not correlated with SCNA score'. I am wondering if there are genes with ASE that are correlated with their SCNA scores. Why not the authors validate the gene with ASE that is top-1 correlated with the SCNA score?

11. On page 8 lines 30-35, the authors mentioned that 'High-impact mutations occur at a substantially higher rate in ASE/non-SCNA samples compared to other categories (Figure 3E) supporting the hypothesis that in genome regions with frequent SCNAs, gene expression is often altered by NMD or other mechanisms in the samples that lack SCNAs'. I am not sure what are the other mechanisms? I am not sure if there is published literature that supports the authors' hypothesis 'Gene expression is often altered by NMD in Neuroblastoma' or if the authors can validate this hypothesis in this paper.

12. I didn't see a full list of ASE genes in neuroblastoma that is predicted by the proposed allele-specific expression algorithm.

13. In the proposed algorithm for ASE analysis, the authors used some constants, such as 3/2 in the equation on page 16 lines 43-45, and 0.5 in the equation on page 16 lines 60-61. These constants should be set as parameters in the proposed algorithm and are better estimated by setting priors in the statistical ASE analysis.

**Authors Response**

**Point-by-point responses to the reviewers’ comments:**

This is an interesting article that analyzes allele specific expression (ASE) in neuroblastoma, in order to find non-coding drivers of cancer. A nice aspect of the paper is that it considers allelic imbalance in the noncancerous setting, as estimated from GTEX expression data, in order to figure out when ASE observed in cancer samples is remarkable. Additionally, the paper gives an approach to detect copy number variation based on allelic imbalance in exome data. The authors find evidence that there are numerous genes with recurrent ASE in neuroblastoma, due either to copy number variation or to other, as yet undetected, alterations. The work showcases the power of ASE to highlight non protein coding driver events in cancer. Overall, the paper should be of an interest to a broad range of scientists working in cancer genomics, computational biology and neuroblastoma.

*We thank the reviewer for their positive feedback and provide detailed responses to their specific comments below.*

The authors should cite PMID 32078798, where Przytycki & Singh have shown that ASE can be used to detect driver genes in the breast cancer context- that work required matched normal RNA-seq data whereas the current manuscript uses GTEX data and additionally gives an approach to detect copy number variants.

*We thank the reviewer for drawing our attention to the highly-relevant paper by Przytycki & Singh. We have added a citation to this paper to the introduction: “To discover genes which are dysregulated by pathogenic events, ASE in disease tissue can be compared to either paired-normal tissue or to a large panel of normal tissues to identify cancer-specific gene dysregulation [22, 23, 30].”*

The overall methodology given in the manuscript seems sound, but its write up needs to be improved substantially. More details are necessary in the section on "Estimating DNA allelic imbalance using exome-seq," and the notation there needs to be very carefully defined.

*We thank the reviewer for the comments and agree that our description of the methodology needed to be improved. We have re-written the methods section extensively to provide more detail and better describe our procedures for estimating DNA imbalance, SCNAs and ASE. We have broken this reviewer comment into subcomments (2A-F) so that we can describe changes that address each point below.*

Some examples: The likelihood does not seem to be written as a likelihood but as a probability.

*We thank the reviewer for this comment. While for continuous random variables likelihoods are not probabilities, we note that for discrete random variables likelihoods are probabilities. We completely agree with the reviewer, however, that our notation for likelihoods needed to be improved, and that it would be more consistent to write the probabilities in using probability mass functions. We have rewritten the equations accordingly. For example, when we first introduce the likelihood equations related to allele-specific read counts we write the following: To model allele-specific read counts we assumed that the reference allele count at heterozygous sites is beta-binomially distributed. The likelihood for the allelic imbalance parameter, 𝑎, and the dispersion parameter, 𝑑, at a single heterozygous site 𝑖 is then defined by:*

*where 𝑝𝑋() is the beta binomial probability mass function; 𝑥𝑅,𝑖 is the observed reference allele count from overlapping reads at site 𝑖; 𝑛𝑖 is the total count of overlapping reads matching the reference or alternate allele at site 𝑖; 𝑝 is the reference allele proportion; 𝑎 is the allelic imbalance parameter and is defined over the range [-0.5,0.5]; 𝑑 is the beta binomial dispersion parameter; and 𝐵() is the beta function. To perform likelihood calculations we used the beta-binomial probability mass function (dbetabinom) provided by the rmutil (1.1.5) package, which is described in the vignette (https://cran.rproject.org/web/packages/rmutil/rmutil.pdf).*

The authors should be more precise with notation (e.g., X_{R, i} is defined but X_{R.tumor, i} is not), and should clarify whether X_{R, i} refers to all reads (tumor and normal) mapping to that site.

*We are now more precise with notation throughout the methods section. We no longer utilize the variable X_{R.tumor, i}, because the calculations are the same for tumor and normal. We now just use a single variable 𝑥𝑅,𝑖 , which we describe as follows: “𝑥𝑅,𝑖 is the observed reference allele count from overlapping reads at site 𝑖"*

When P_{BB} is first used, the manuscript should state that this is the probability using beta binomial.

*We no longer use the notation P_{BB}. We now make it clear that the likelihood calculations utilize the beta binomial distribution with the statement “𝑝𝑋() is the beta binomial probability mass function” and by providing the full formula for the beta binomial probability mass function.*

It's not clear why the formula for L(X_{R, i}|parameters} is given considering both tumor and normal together, when ML estimates are likely done separately for tumor and normal.

*Thank you for pointing this out. We previously did the ML estimates jointly even though it was not necessary to do so. We now perform the estimates separately for tumor and normal samples (the results are the same) and simplify the description in the methods accordingly.*

Please give more details on how dispersion parameter is estimated - Is this over sites in the bin? Are all sites assumed to be independent?

*We now explain that we estimate the dispersion parameter over all heterozygous sites. In the case of DNAimbalance this is across all heterozygous sites genome-wide as we describe in the relevant part of the methods: We estimate a single dispersion parameter across all heterozygous sites genome-wide. This is accomplished by fixing 𝑎 to 0 and finding the value of 𝑑 that maximizes the total likelihood across all heterozygous sites in either a normal or a tumor sample:*

*𝑑̂ = argmax𝑑∏ ℒ𝑖(𝑎 = 0, 𝑑|𝑥𝑅,𝑖 , 𝑛𝑖)*

*In the case of ASE estimated from RNA-seq reads, the dispersion is estimated across all heterozygous sites within exons as we now describe in the methods:*

To find the maximum likelihood estimate of 𝑑 and 𝜖𝑆 , we fix 𝑎 to 0 and optimize 𝑑 and 𝜖𝑆 over all heterozygous sites overlapping exons:

𝑑̂, 𝜖̂𝑆 = argmax𝑑,𝜖𝑠∏ ℒ𝑖(𝑎𝑖 , 𝑑, 𝜖𝑆|𝑥𝑅,𝑖 , 𝑛𝑖 , 𝜖𝐺,𝑖)

Are all sites assumed to be independent?

*See response to R1 comment 3 below.*

What types of dispersion values are estimated?

*The dispersion parameter describes the overdispersion in the observed read counts for the reference allele, which exceeds what is expected from binomial sampling alone. That is, we treat the beta binomial as an overdispersed binomial distribution. We use a parameterization of the beta binomial distribution that has parameters for mean (proportion) and dispersion. To make the meaning of the parameters more explicit, we now provide the probability mass function in the paper:*

The formula in the middle of page 15 appears to be missing a multiplication sign.

*We thank the reviewer for pointing this out. We have re-written this part of the methods in response to other comments and the old formula, which was missing the multiplication sign, is no longer present.*

The model assumes independence over sites, this may not be valid as nearby sites are correlated; the authors should comment on this.

*For estimating DNA imbalance (and dispersion) from exome-seq reads we assume independence between sites. We think this is only a small problem since most heterozygous sites within a sample are spaced far apart, most reads overlap only a single heterozygous site, and the SCNA estimates combine information across multiple bins, each of which contains multiple variants. For estimating ASE from RNA-seq reads we use WASP to obtain allelic counts. WASP uses a sampling procedure to remove the dependence between nearby SNPs, which we now describe in the methods: We filtered RNA-seq reads for mapping bias and obtained allele specific read counts at heterozygous positions using WASP [73]. WASP uses random sampling to ensure that allele counts at nearby heterozygous sites are independent. Specifically, when a read overlaps multiple sites, the allelic count is incremented at only one of the sites, which is selected randomly.*

For section on estimating allelic expression:

*We have broken this comment into subcomments 4A and 4B so that each can be addressed separately.*

What is n in the equation for epsilon_s? Please clarify where this comes from: "1/3rd of sequencing errors will be counted as alternate or reference alleles".

*In the previous version of the manuscript, n was the total number of heterozygous sites (we estimated epsilon_s across all heterozygous sites). The previous version of the paper used the count of “other” alleles (not matching either reference or alternate allele) to estimate the sequencing error rate. The 1/3 came from the assumption that a sequencing error can generate 3 possible base changes away from the true allele, however 1/3 of these changes would not be counted as an ‘other’ allele because they would match the alternate (or reference) allele for the variant. In the new version of the manuscript, we no longer estimate epsilon_s in this way, and we no longer include the equation with an ‘n’ or a 1/3. As described in response to Reviewer 2 comment 13 below we now treat epsilion_s as a parameter and estimate it by maximum likelihood.*

What is little p? Where is episilon_g defined? Additional text that helps the reader understand the methodology is necessary.

*The little p is the reference allele proportion parameter for the beta binomial distribution. This is now defined clearly in the text where we describe the likelihood equation: “𝑝 is the reference allele proportion. epsilion_g is the genotyping error probability. We have updated the definition provided in the text to make it clear that each heterozygous site i has its own genotyping error probability that is computed from GATK’s genotype quality score. We also provide substantially more context describing why we use the genotyping error probability: Genotyping errors can create a false signal of allelic imbalance. For the SCNA analysis above, DNA imbalance is estimated from many heterozygous sites within each bin and then estimates from multiple bins are combined with circular binary segmentation. Since many heterozygous sites are used for this analysis, genotyping errors can be ignored without a major effect. However, when estimating ASE for a single gene we often utilize a small number of heterozygous sites, so it is desirable to account for genotyping errors. To control for genotyping errors, we calculate the genotyping error rate, 𝜖𝐺,𝑖 , for each heterozygous site 𝑖 directly from the genotype quality (GQ) scores provided by GATK:*

**Reviewer #2:**

In this paper, Sen et al. analyzed allele-specific expression (ASE) in 96 high-risk neuroblastoma tumors to discover genes with cis-acting mutations that alter the dosage. The authors discovered that many genes exhibit ASE in copy neutral samples and are enriched for mutations that cause nonsense-mediated decay and 24 genes with reduced expression in stage 4 disease that have neuroblastoma-specific ASE that is independent of SCNAs. The authors generated some interesting hypotheses based on the ASE of genes, however, most of their hypotheses are not convincing. I have some technical questions and concerns about the proposed algorithm for ASE analysis and the in-vitro experiments. Blow, I list my specific comments.

*We thank the reviewer for their constructive criticism. We address their specific concerns related to the hypotheses and technical details below.*

Have the authors tried the haplotype phase algorithm in exome-seq data? As the proposed model was based on a 50% probability of SNPs on the same haplotype.

*We tried out three phasing algorithms: HapCUT2, phASER and Eagle v2.4 ( Michigan Imputation Server). HapCUT2 and phASER are read-based phasing methods that can be used to phase both somatic mutations and germline variants. Eagle is a reference population-based method for phasing germline variants. For each method we computed the percentage of heterozygous variants which can be phased from the exome-seq data for each sample (see Figure).*

*We observed that the read-based methods (HapCUT2 and phASER) can phase ~30% of the heterozygous variants. Eagle phases anywhere between 6% and 90% of variants. We think the samples that are phased poorly by Eagle are likely those with a high somatic mutation burden. We have modified our algorithm for ASE and DNA allelic imbalance information so that it takes into account all possible phasings of heterozygous variants. We feel this is preferable to relying on output from phasing algorithms, because in all tumors a substantial number of variants are unable to be phased. Our new description of our likelihood calculations is as follows:*

*After estimating the dispersion parameter, we estimate the allelic imbalance and compute the likelihood for bins. In the case of DNA allelic imbalance, a bin consists of 20 consecutive exons as described above. To estimate the imbalance of a bin, we combine information across all of the heterozygous sites within the bin. Since we do not know the phasing of the alleles, we consider all possible phasings (i.e. haplotype configurations), when computing the likelihood. Under the assumption that all phasings are equally probable, the likelihood of the parameters for bin 𝐵 is:*

*where 𝐻𝐵 is the set of all possible phasings (i.e. hapolotype configurations) for bin 𝐵. A phasing is defined by a vector of 0s and 1s, with each element corresponding to a heterozygous site. Element 𝑖 of the phase vector is set to ℎ𝑖 = 0 if the reference allele for site 𝑖 is on ‘chromosome A’ and set to ℎ𝑖 = 1 if the reference allele for site 𝑖 is on ‘chromosome B’. ‘Chromosome A’ is defined as the chromosome that carries the reference allele for the first heterozygous site in the bin. In total there are ‖𝐻𝐵‖ = 2 𝑚−1 possible phasings, where 𝑚 is the number of heterozygous sites within the bin. While the number of phasings grows exponentially with 𝑚, the number of heterozygous sites in the bin, the likelihood for the bin can be computed efficiently in linear time using dynamic programming:*

*where 𝑇 is an array of length 𝑚 that is used to compute the cumulative likelihood.*

*We have applied this modified method to our dataset, and we found that it yielded very similar results to our previous algorithm. For example, in the previous version of the manuscript we observed 1,049 genes with recurrent, neuroblastoma-specific ASE, 68% of which lie within common SCNA regions. In our updated results we find 1,043 genes with recurrent neuoblastoma-specific ASE, 65% of which are within common SCNA regions.*

Were the authors being able to check the allele imbalance methylation on promoters of allelic imbalance genes?

*We thank the reviewer for this comment and agree that being able to test allele imbalance at promoter methylation sites for ASE genes would be a great addition to the paper. Unfortunately this is not possible because our methylation data comes from a microarray platform rather than from bisulfite sequencing. Specifically, we used the pre-computed methylation measurements for Human Methylation 450K array available through Therapeutically Applicable Research to Generate Effective Treatments (TARGET). We are not aware of a method that can estimate allele-specific methylation from this type of data.*

In my opinion, it is a bit risky to keep SNP sites with sequence errors incorporated in the model as you assigned an equal probability of sequence errors to reference or alternative.

*We agree with reviewer and acknowledge that it is risky to keep SNPs with high ‘other’ allele counts. In our revised analysis, we now remove all heterozygous sites with greater than two reads containing ‘other’ alleles prior to performing our ASE analysis. We now describe this in the methods section:*

*We observed that at most heterozygous sites overlapping RNA-seq reads only match the reference or alternate alleles. However, some sites also have reads that match neither allele, which we refer to as ‘other’ reads. ‘Other’ reads may reflect sequencing errors or mis-mapped reads so we removed all heterozygous sites where the ‘other’ read count was greater than two prior to ASE analysis.*

What was the way the authors used to call mutation in promoter regions and other non-coding regions with exome-seq?

*We thank the reviewer for raising this issue. In our study we do NOT call mutations in promoter regions or other non-coding regions. Rather we can infer the effects of cis-acting mutations by observing allele-specific expression (ASE) of genes. ASE is estimated from RNA-seq reads overlapping heterozygous sites in the exons of genes, however we do not expect most of these heterozygous sites to actually be causal. Rather these heterozygous sites provide a means to readout ASE, which is a signal that there is some cis-acting genomic alteration that affects the expression of one allele. In many cases we do not know the identity of the cis-acting alteration that causes ASE, although sometimes we can infer that it is caused by SCNA or NMD To make these issues much more clear to readers of our paper, we have revised several parts of the manuscript,.*

*We added a sentence to the intro a call out to a new Figure 1A to describe these issues better:*

*This makes ASE a powerful tool for revealing genes that are affected by cis-acting mutations, including noncoding regulatory mutations that affect sequences such as promoters, enhancers, and insulators as well as protein-coding or splicing mutations that result in nonsense-mediated decay (NMD) (Figure 1A). Another advantage of ASE is that it is detectable even when the identity of the pathogenic variants causing dysregulation are unknown and it can reveal the effects of rare germline or somatic mutations [22, 23]. Thus, ASE is a powerful tool for the identification of genes with altered gene dosage due to cis-acting genome alterations.*

*The new Figure 1A describes the different types of mechanisms that can cause ASE.*

*In addition, we have added the following sentences to the discussion to indicate that we often don’t know the identity of the causal mutations and that whole-genome sequencing could potentially help discover some of them:*

*In some samples, ASE in the absence of SCNAs can be attributed to mutations that cause NMD, however in other samples the cause is unknown. In these cases, the cause of ASE could potentially be revealed by future studies. For example whole genome sequencing and analysis of non-coding mutations near NB-ASE genes could illuminate cis-acting regulatory mutations that cause ASE.*

I am not sure what are "ADRNLG" and "WHLBLD" in figures 1B and 1C?

*Thank you for pointing out that these terms were not defined in the manuscript. We have replaced these abbreviations with “adrenal gland” and “whole blood” (or “blood”) to correct this issue.*

On page 7 lines 27-29, the authors mentioned that 'We further examined the relationship between ASE and SCNAs for specific genes in the chromosome 1p and 3p deletion regions that are hypothesized to be tumor suppressors.', however, not sure why ASE and SCNAs for specific genes in chromosome 1p and 3p deletion regions are tumor suppressors, would the authors clarify more?

*We thank the reviewer for pointing out that this was not clear. Prior studies have hypothesized that specific genes within the Chromosome 1p and 3p deletion regions are tumor suppressors. We are interested in examining ASE of these genes to see if some samples show ASE in the absence of a deletion, indicating that there are multiple genomic alterations that affect their expression. We have revised and added text to explain the motivation and provide additional background as follows:*

*The chromosome 1p, 3p and 11q deletion regions are hypothesized to contain tumor suppressor genes, however the identities of the tumor suppressors are difficult to determine because the deletions are large and contain hundreds of genes. We reasoned that in the absence of large deletions tumor suppressors within these regions may be affected by other types of genome alterations that affect dosage, and that the effects of these alterations would be detectable by ASE. We examined the relationship between ASE and SCNAs for two genes, IP6K2 and KIF1B, which have been previously identified as potential tumor suppressors located within the chromosome 3p and 1p deletion regions. Knockdown of IP6K2 impairs apoptosis in colorectal cancer cells [44] and its deletion or low expression is associated with adverse clinical outcomes in aerodigestive tract carcinoma and breast cancer [45, 46]. Overexpression of KIF1B in neuroblastoma cell lines causes apoptotic cell death and its knockdown enhances tumor formation in mouse models [33]. In the case of IP6K2, we found that every sample with significant ASE also has a high SCNA score (Figure 2E, right panel), indicating that ASE of IP6K2 is solely attributable to overlapping chromosome 3p deletions. The pattern exhibited by KIF1B is different. While ASE of KIF1B is correlated with SCNA score (Spearman’s rho = 0.68, FDR corrected pvalue = 2.4e-07), several samples have strong ASE in the absence of a chromosome 1p deletion (SCNA score ≤ 0.09) (Figure 2E). Thus, in some samples, ASE of KIF1B is caused by factors other than large-scale SCNAs. Several other putative tumor suppressors in the chromosome 1p or 11q deletion regions including CHD5 [15, 47], UBE4B [13], CADM1 [48] and ATM [49], have patterns that are similar to KIF1B, where a subset of samples exhibit strong ASE in the absence of deletions (Supplementary Figure S4). These results indicate that both SCNA and non-SCNA genome alterations affect the expression of these genes.*

On page 7 line 47, the authors mentioned that 'We hypothesized that some ASE events are caused by nonsense-mediated decay (NMD) introduced by mutations that create premature stop codons including frameshifting indels, nonsense mutations, stop-loss mutations, or mutations that alter splicing', which is very interesting, however, there is no convincing evidence to support this hypothesis.

*We thank the reviewer for pointing out that the nature of our hypothesis was not clear. We note that it is not our hypothesis that these types of mutations should cause nonsense-mediated decay NMD, as this is already wellestablished by prior work. In addition, it has been previously observed that genes carrying heterozygous premature stop codons exhibit ASE, as a signature of NMD. For example, this has been observed in studies by Lappalainen et al., 2013; MacArthur et al., 2012; and Montgomery et al., 2011. Our hypothesis is that NMDcausing mutations are a common mechanism for ASE in neuroblastoma in addition to SCNAs. To make this more clear in the revised manuscript we have 1) cited the relevant literature, 2) clarified the specific hypotheses in the text; 3) refocused the analysis of ‘high-impact’ mutations on ‘stop-gain’ mutations (which have the clearest connection with NMD); 4) provided a graphic that explains the connection between ASE and NMD in Figure 1A (see above in response to Comment 4). The re-worked section of the manuscript is as follows:*

*Previous studies have demonstrated that ASE can be caused by nonsense-mediated decay (NMD) [53-55], an evolutionarily-conserved mechanism that degrades transcripts with premature termination codons [50][51, 52] (Figure 1A). We hypothesized that genes that exhibit ASE in neuroblastoma in the absence of SCNAs may contain mutations that cause NMD. To identify somatic mutations that are likely to cause NMD, we analyzed paired tumor-normal exome-seq data with Variant Effect Predictor (VEP), which collectively labels missense, frameshift, and nonsense mutations that are likely to cause NMD as ‘high-impact’. We identified 12,122 unique high-impact mutations in the 96 tumor samples, 886 of which are located within 490 NB-ASE genes. Most of these mutations (788 out of 890) are stop-gain mutations (Figure 3A and Supplementary Table 6) and map to 452 NB-ASE genes. To determine if stop-gain mutations are enriched within NB-ASE genes, we examined their frequency in three different gene sets: a) NB-ASE genes, b) randomly selected genes with at least one somatic mutation, and c) genes with ASE observed in both neuroblastoma and normal tissues. To create a null distribution, we sampled 100 genes from each gene set 500 times and counted the number of genes carrying stop-gain mutations each sampling iteration. A substantially greater number of NB-ASE genes carry stop-gain mutations, indicating that NMD is an important driver of neuroblastoma-specific ASE (Figure 3B). We observed that many genes with correlated ASE and SCNA scores also contain stop-gain mutations in some samples (Figure 3C), leading us to hypothesize that NMD is an important mechanism that alters gene dosage in samples lacking SCNAs. To test this hypothesis, we partitioned neuroblastoma samples for each gene into three categories: non-ASE samples; ASE samples with SCNAs (ASE_SCNA); and ASE samples without SCNAs (ASE_non-SCNA) (Figure 3D). We then calculated the rate of stop-gain mutations across all genes and samples in each of the three categories. To generate a null distribution of rates that controls for gene lengths and mutation rate heterogeneity, we permuted the category labels for each gene 1000 times. This analysis revealed that stop-gain mutations occur at a substantially higher rate in ASE_non-SCNA samples compared to other categories (Figure 3E). This supports the hypothesis that gene expression is often altered by NMD-causing mutations in the samples that lack SCNAs.*

In Figure 6, it is unclear that if PTPRH knockdowns (KDs) in SK-N-BE (2) and SK-N-SH cells are allelic specific or not? If PTPRH knockdown is not in an allelic-specific manner, not sure how the in-vitro experiments validate the functions of ASE of PTPRH. The title of this paper is "Allele-specific expression links diverse cis-regulatory mutations to recurrent gene dysregulation in high-risk neuroblastoma", I am not sure how the ASE of PTPRH links to cis-regulatory mutations in high-risk neuroblastoma. There is no whole genome sequencing data in this paper, not sure how cis-regulatory mutations were identified. I would suggest the authors changing the title to better fit the content of this paper.

*We thank the reviewer for this comment as it reveals some concepts that could have been better explained in the paper. The purpose the ASE analysis is to identify genes with dosage that is altered by cis-acting genome alterations. We do not think that the ASE intrinsically has a functional effect, rather it is the change in gene expression that matters to the cell. We have added text and a new Figure 1A to better explain the purpose of the ASE analysis as described in response to Reviewer 2 Comment 4 above.*

*In the case of PTPRH we demonstrate in Figure 5D that gene expression of PTPRH is negatively correlated with ASE. Therefore, the ASE of PTPRH appears to be a signal of cis-acting genomic alterations that decrease the expression of one allele of PTPRH. Based on these observations, we believe that knockdown of PTPRH is an appropriate experiment to test the function of PTPRH.*

*We agree with the reviewer that the title of the paper could better reflect the content of the paper. We have changed the title of the paper to “Allele-specific expression reveals genes with recurrent cis-regulatory alterations in high-risk neuroblastoma.”*

There is no in-vivo data to validate the function of PTPRH with ASE. It is not convincing if there is no in-vivo validation of PTPRH KDs, which is not difficult to perform.

*We thank the reviewer for this comment and acknowledge that in-vivo models could provide additional evidence for the function of PTPRH in neuroblastoma. While in vivo data would be beneficial, we feel that these experiments would involve substantial time and effort and would far exceed the amount of time we have been granted for revisions.*

*In addition, we feel that the results we present in the study collectively provide good evidence that PTPRH expression is important for neuroblastoma:*

*o ASE of PTPRH is enriched in neuroblastoma tumors suggesting that PTPRH is a target of cisacting mutations in these tumors. (Figure 5A)*

*o In a separate larger dataset (498 tumors) which contains both high and low risk patients, PTPRH shows the lowest expression in high-risk stage 4 tumors (Figure 5E) and is significantly correlated with event-free survival (Figure 5F).*

*o PTPRH knockdown in two neuroblastoma cell lines in increases cell confluence (Figure 6E).*

*o PTPRH knockdown in the SK-N-SH neuroblastoma cell line decreases cell migration (Figure 6F).*

*In vivo experiments would of course make these arguments even stronger, however given the scope of our paper we feel they are best left for future studies. To acknowledge the lack of in-vivo experiments we have added the following sentence to discussion:*

*In our study we found that: a) PTPRH exhibits recurrent ASE in neuroblastoma, b) low expression of PTPRH is associated with adverse patient outcomes, and c) knockdown of PTPRH increases proliferation and wound healing in neuroblastoma cell lines. Collectively, these observations suggest that PTPRH functions as a tumor suppressor in high-risk neuroblastomas. Confirmation that PTPRH acts as a tumor suppressor will require in vivo experiments that are beyond the scope of this study.*

On page 10 line 24, the authors mentioned that 'ASE of PTPRH is not correlated with SCNA score'. I am wondering if there are genes with ASE that are correlated with their SCNA scores. Why not the authors validate the gene with ASE that is top-1 correlated with the SCNA score?

*We thank the reviewer for raising this point as it reveals some aspects of the paper that could have been written more clearly. We would like to clarify that the major goal of the paper is not to identify ASE that is associated with SCNA score. SCNAs are the most common genetic alteration observed in neuroblastomas and it was important in our study to determine what fraction of ASE was attributable to these large-scale SCNA events. As such, we do examine the correlation of ASE and SCNA as described on Pg 7 (“Under an FDR of 10%, 65% (684 out of 1,043) of NB-ASE genes are significantly correlated with SCNAs and of these, 59% (401 out 684) are located on the chromosomes with the most frequent SCNAs (chromosomes 1, 3, 11 and 17).” However, we feel in this paper it is actually more interesting to examine genes that have ASE in the absence of SCNAs because this can reveal other mechanisms for altering the dosage of genes, beyond the wellestablished SCNAs. In addition, a challenge with SCNAs is that they typically span hundreds of genes, making it difficult to identify the important genes within these regions. We feel that ASE analysis provides an alternative means to identify genes with altered dosage, including those that have altered dosage in the absence of SCNAs. To clarify these issues in the paper we have added or edited the following text:*

*Pg 7: “The chromosome 1p, 3p and 11q deletion regions are hypothesized to contain tumor suppressor genes, however the identities of the tumor suppressors are difficult to determine because the deletions are large and contain hundreds of genes. We reasoned that in the absence of large deletions tumor suppressors within these regions may be affected by other types of genome alterations that affect dosage, and that the effects of these alterations would be detectable by ASE.”*

*Pg 8: “Thus, in some samples, ASE of KIF1B is caused by factors other than large-scale SCNAs. Several other putative tumor suppressors in the chromosome 1p or 11q deletion regions including CHD5 [15, 47], UBE4B [13], CADM1 [48] and ATM [49], have patterns that are similar to KIF1B, where a subset of samples exhibit strong ASE in the absence of deletions (Supplementary Figure S4). These results indicate that both SCNA and non-SCNA genome alterations affect the expression of these genes.” Pg 12: “Neuroblastoma genomes contain a surprisingly large number of genes with recurrent ASE; however, the majority of ASE events can be attributed to SCNAs which are well-characterized and common genomic alterations in neuroblastoma that typically span tens of megabases.” “Our ASE analysis reveals that, in some samples, genes within recurrent SCNA regions are dysregulated by non-SCNA events. Non-SCNA ASE events are present in genes which have been previously described as putative tumor suppressors and studied in the context of recurrent SCNAs including KIF1B, PLEKHG5, UBE4B, CHD5, CADM1, and ATM. With larger sample sizes, ASE could potentially be utilized to distinguish passenger genes from driver genes within recurrent SCNA regions. In some samples, ASE in the absence of SCNAs can be attributed to mutations that cause NMD, however in other samples the cause is unknown. In these cases, the cause of ASE could potentially be revealed by future studies. For example whole genome sequencing and analysis of non-coding mutations near NB-ASE genes could illuminate cis-acting regulatory mutations that cause ASE.”*

On page 8 lines 30-35, the authors mentioned that 'High-impact mutations occur at a substantially higher rate in ASE/non-SCNA samples compared to other categories (Figure 3E) supporting the hypothesis that in genome regions with frequent SCNAs, gene expression is often altered by NMD or other mechanisms in the samples that lack SCNAs'. I am not sure what are the other mechanisms? I am not sure if there is published literature that supports the authors' hypothesis 'Gene expression is often altered by NMD in Neuroblastoma' or if the authors can validate this hypothesis in this paper.

*We thank the reviewer for this comment. In this study we have tested three mechanisms which may cause ASE: SCNAs, NMD-causing mutations and DNA methylation. Additional mechanism may include splicing mutations, and non-coding mutations which impact regulatory regions like promoters, enhancers, and insulators. To illustrate some of the mechanisms that cause ASE, we have added Figure 1A (see above in response to Reviewer 2 Comment 4). To clarify the NMD hypothesis we made substantial changes to the manuscript as described in response to Reviewer 2 Comment 7 above.*

I didn't see a full list of ASE genes in neuroblastoma that is predicted by the proposed allelespecific expression algorithm.

*Complete results of the ASE analysis were provided in Supplementary Table 1. We were unable to upload this large table into the online manuscript submission system, but we provided it separately to the editor. Perhaps the reviewer did not receive it?*

In the proposed algorithm for ASE analysis, the authors used some constants, such as 3/2 in the equation on page 16 lines 43-45, and 0.5 in the equation on page 16 lines 60-61. These constants should be set as parameters in the proposed algorithm and are better estimated by setting priors in the statistical ASE analysis.

*Thank you for the suggestion. We have modified our algorithm so that the sequencing error, 𝜖𝑆 is estimated by maximum likelihood without any constants assumed. This is described in the revised methods section as follows:*

*When a genotyping error occurs, a heterozygous site can be homozygous reference (0/0) or alternate (1/1), and we assume these two possibilities are equally likely. When there is a genotyping error and the sample is homozygous, all sequencing reads that come from one of the two alleles must arise due to sequencing or mapping errors. We use the parameter 𝜖𝑆 to describe the frequency of these sequencing errors. The likelihood of the parameters for a single heterozygous site is then:*

*where, as described above, 𝑝𝑋() is the probability mass function for the beta binomial distribution. To find the maximum likelihood estimate of 𝑑 and 𝜖𝑆 , we fix 𝑎 to 0 and optimize 𝑑 and 𝜖𝑆 over all heterozygous sites overlapping exons:*

*For optimization we used the L-BFGS-B algorithm implemented by the ‘optim’ function provided by the stats package in R-4.0.1. Due to changes in how we treat the phasing of alleles (described in response to Reviewer 2 Comment 1 above) we no longer use the 0.5 constant. We found that the changes to the algorithm did not substantially change the overall results, as described in Reviewer 2 Comment 1 above.*

**Second round of review**

**Reviewer 2**

1. The authors mentioned in method part that bin size for ASE analyses is 20 consecutive exons, according to a benchmark paper evaluating different phasing algorithms, that EAGLE2 is able to correctly phase 44 to 64 consecutive SNPs and around 2% of haplotype blocks contain errors based on their sample size (https://doi.org/10.1186/s12859-019-3095-8). I guess bin sizes by 20 consecutive exons might have a very wide range depending on the size of genes. I recommend the authors carefully select bin sizes (e.g., based on heterozygous SNPs or fixed length instead of exon sizes) if incorporating the phase info in their algorithm.

2. If the authors did not conduct mutation calling in non-coding regions. I highly recommend the authors perform WGS analysis on non-coding regions if the matched WGS data is available. This can be helpful to explain why the authors raise four mechanisms in Fig.1A. In addition, including non-coding regions mutations help us to know if NMD exclusively causes ASE or NMD and other mechanisms work collectively to change genes dosage.

3. There are several published papers (PMID:32025019, PMID:34731645, and PMID:32078798) that studied allelic imbalance at the DNA and RNA levels and the correlation between allele-specific expression and somatic CNV and SNV in cancer. I'm not sure what new concepts or advancements are introduced in this paper.

4. Since in-vivo results can be different from in-vitro results, I still strongly recommend the authors validate the function of PTPRH with ASE in-vivo.

**Authors Response**

**Point-by-point responses to the reviewers’ comments:**

Reviewer #2: Major Comments:

1. The authors mentioned in method part that bin size for ASE analyses is 20 consecutive exons, according to a benchmark paper evaluating different phasing algorithms, that EAGLE2 is able to correctly phase 44 to 64 consecutive SNPs and around 2% of haplotype blocks contain errors based on their sample size (https://bmcbioinformatics.biomedcentral.com/articles/10.1186/s12859-019-3095-8). I guess bin sizes by 20 consecutive exons might have a very wide range depending on the size of genes. I recommend the authors carefully select bin sizes (e.g., based on heterozygous SNPs or fixed length instead of exon sizes) if incorporating the phase info in their algorithm.

*We thank the reviewer for their comment and would like to clarify that the bins of 20 exons are not used to estimate allele-specific expression (ASE). These bins are only used to estimate DNA imbalance and somatic copy number alterations. We estimate ASE for each gene by using all of the heterozygous sites within the exons of the gene being considered.*

*To make this clear we have added additional sentences to the results and methods section:*

*Results section: “To estimate ASE in these samples, we implemented a statistical model that utilizes allele-specific read counts at heterozygous sites within the exons of genes…”*

*Methods section (pg 17): “In the case of DNA allelic imbalance, a bin consists of 20 consecutive exons as described above. In the case of RNA allelic imbalance, we utilize the set of heterozygous sites within the exons of the gene being considered as described below.”*

*Methods section (pg 18): “However, when we calculate ASE for a gene, we only consider heterozygous sites within the exons of the gene.”*

2. If the authors did not conduct mutation calling in non-coding regions. I highly recommend the authors perform WGS analysis on non-coding regions if the matched WGS data is available. This can be helpful to explain why the authors raise four mechanisms in Fig.1A. In addition, including non-coding regions mutations help us to know if NMD exclusively causes ASE or NMD and other mechanisms work collectively to change genes dosage.

*We agree with the reviewer that analysis of matched WGS data would add to the paper. Unfortunately, matched WGS data is not available for the TARGET neuroblastoma samples that we used in the paper. The TARGET consortium performed WGS of neuroblastoma samples using the defunct Complete Genomics platform. We contacted the authors of the TARGET neuroblastoma papers and we were told that mutation calls and usable WGS data for these samples does not exist.*

3. There are several published papers (PMID:32025019, PMID:34731645, and PMID:32078798) that studied allelic imbalance at the DNA and RNA levels and the correlation between allele-specific expression and somatic CNV and SNV in cancer. I'm not sure what new concepts or advancements are introduced in this paper.

*We thank the reviewer for their comment. While allele-specific analysis of RNA and DNA are not new concepts, to our knowledge our study is the first to use this approach to study gene dysregulation in neuroblastoma.*

*Furthermore, our method of estimating ASE and DNA allele imbalance has several improvements which distinguishes it from the following prior studies.*

*a) PMID:32025019 (PCAWG Transcriptome Core Group et al, 2020): this paper used phased genotypes from WGS and estimated allele-specific expression for genes by summing the read counts on the respective haplotypes to obtain gene level haplotype-specific read counts. They then used a binomial test against an expected read ratio of 0.5 to obtain nominal P values.*

*b) PMID:32078798 (Przytycki et al, 2020): this paper also used a binomial distribution to compute p-values for each heterozygous site and then combined the p-values across heterozygous sites using Fisher’s method.*

c) PMID:34731645 (Ciani et al, 2021) employs an allele-specific copy number analysis (asCN) pipeline to discover copy gain, loss and LOH regions and uses a linear model to predict the expression level of a gene based on the total number of copies and the presence of LOH.

*We have added these references to the paper in the following sentence:*

*“To discover genes which are dysregulated by pathogenic events, ASE in disease tissue can be compared to either paired-normal tissue or to a large panel of normal tissues to identify cancer-specific gene dysregulation [22, 23, 30, 31].”*

*Our method has several improvements over the methods of these papers:*

*a) Our method corrects reads for reference mapping bias which causes false positives (Degner et al 2009, Bioinformatics),*

*b) Our method uses a beta binomial distribution and accounts for overdispersion in read counts. The binomial test does not account for overdispersion of read counts and overestimates the number of allele-specific events (Chen et al, 2016, Nat Communications )*

*c) Our method takes into account the possibility of sequencing and genotyping errors which can cause false positives.*

*d) Our method can combine information across SNPs even when haplotype phasing information is unavailable.*

4. Since in-vivo results can be different from in-vitro results, I still strongly recommend the authors validate the function of PTPRH with ASE in-vivo.

*We acknowledge that in-vivo models could provide different conclusions from in-vitro experiments. However, we feel that in-vivo experiments are beyond the scope of this study. We note this limitation at the end of the discussion section: “Confirmation that PTPRH acts as a tumor suppressor will require in vivo experiments that are beyond the scope of this study.”*
